# Role of Dipolar Interactions on the Determination of the Effective Magnetic Anisotropy in Iron Oxide Nanoparticles

**DOI:** 10.1002/advs.202203397

**Published:** 2022-12-12

**Authors:** Pelayo García‐Acevedo, Manuel A. González‐Gómez, Ángela Arnosa‐Prieto, Lisandra de Castro‐Alves, Yolanda Piñeiro, José Rivas

**Affiliations:** ^1^ NANOMAG Laboratory Applied Physics Department Materials Institute (iMATUS) Universidade de Santiago de Compostela Santiago de Compostela 15782 Spain

**Keywords:** dipolar interactions, iron oxide nanoparticles, magnetic anisotropy, magnetic hyperthermia, silica coating

## Abstract

Challenging magnetic hyperthermia (MH) applications of immobilized magnetic nanoparticles require detailed knowledge of the effective anisotropy constant (*K*_eff_) to maximize heat release. Designing optimal MH experiments entails the precise determination of magnetic properties, which are, however, affected by the unavoidable concurrence of magnetic interactions in common experimental conditions. In this work, a mean‐field energy barrier model (Δ*E*), accounting for anisotropy (*E_A_*) and magnetic dipolar (*E_D_*) energy, is proposed and used in combination with AC measurements to a specifically developed model system of spherical magnetic nanoparticles with well‐controlled silica shells, acting as a spacer between the magnetic cores. This approach makes it possible to experimentally demonstrate the mean field dipolar interaction energy prediction with the interparticle distance, d_ij_, *E_D_* ≈ 1/d_ij_^3^ and obtain the *E*_A_ as the asymptotic limit for very large d_ij_. In doing so, *K*_eff_ uncoupled from interaction contributions is obtained for the model system (iron oxide cores with average sizes of 8.1, 10.2, and 15.3 nm) revealing to be 48, 23, and 11 kJ m^−3^, respectively, close to bulk magnetite/maghemite values and independent from the specific spacing shell thicknesses selected for the study.

## Graphical abstract

This work reports the accurate determination of the anisotropy constant by identifying and discriminating the dipolar interaction effects arising in dense magnetic nanoparticle (MNP) assemblies based on a model system of iron oxide MNPs coated by a SiO_2_ shell of controlled thickness, which is of particular interest for maximizing the heat release of MNPs through Néel relaxation in magnetic hyperthermia.

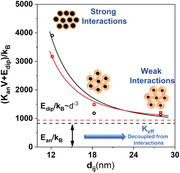

## 1 Introduction

Magnetic nanoparticles (MNPs) have been one of the most studied nanomaterials in recent decades, due to their versatility for applications in fields like biomedicine^[^ ^1^ ^]^ or technology.^[^ ^2^ ^]^ The use of MNPs in magnetic hyperthermia processes is especially important in healthcare therapies. In these treatments, MNPs, are exposed to an alternating magnetic field, absorb magnetic energy, and transform it into thermal energy through relaxation processes, consequently providing local heating to targeted cancer cells^[^ ^3^ ^]^ and hard‐to‐reach tumors.^[^ ^4^ ^]^ Generally, Néel (*τ*_N_) and Brown (*τ*_B_) relaxations^[^ ^5^ ^]^ drive these processes and depend, respectively, on the volume and effective anisotropy constant (*K*_eff_) of the MNPs and the properties of the surrounding media in which the MNPs are dispersed (viscosity and hydrodynamic volume). In real applications, it is not possible to modify crucial parameters governing the performance of magnetic hyperthermia, like the viscosity existing in certain biological entities^[^ ^6^ ^]^ or the impossibility of MNPs to undergo physical rotation when anchored within solid matrices (implantable devices, scaffolds),^[^ ^7^ ^]^ or introduced into cells or tissues.^[^ ^8^ ^]^ Therefore, with a negligible contribution of the Brown relaxation, the enhancement of the hyperthermia performance requires the optimization of the Néel relaxation parameters that remain unaffected by the biological microenvironment.^[^ ^9^, ^10^ ^]^ Hence, in recent years, a great deal of effort has been made to design iron oxide‐based MNPs such as nickel‐substituted zinc ferrite,^[^ ^11^ ^]^ core/shell iron oxide^[^ ^12^, ^13^ ^]^ or maghemite MNPs,^[^ ^14^ ^]^ aiming to maximize heat generation by tailoring *K*_eff_. This strategy requires the precise determination of *K*_eff_, to correctly predict the magnetic response of MNPs^[^ ^15^ ^]^ in magnetic hyperthermia applications.^[^ ^16^ ^]^ Experimental conditions for the magnetic characterization of MNP systems, where samples are dried and closely encapsulated in hermetical holders, supresses mechanical rotation and restrict magnetic reversal only to the Néel mechanism, facilitating *K*_eff_ determination. However, the unavoidable concurrence of factors, such as sample polydispersity or magnetic interactions,^[^ ^17^ ^]^ causes the imprecise determination of the magnetic properties, preventing a precise interpretation of the same and generating controversies in the field.

Thermal activation of magnetization relaxation between consecutive minimum energy configurations, for non‐interacting MNPs, is achieved by surmounting the energy barrier (ΔE),  Δ*E* = *E*_2_ − *E*_1_, between two minima. When the thermal fluctuations are small compared to ΔE, the probability of switching is governed by the Arrhenius‐Néel law^[^ ^18^ ^]^:

(1) $$\tau_{N}=\tau_{0}\exp\left(\frac{\Delta E}{k_{B}T}\right)$$

The well‐established static Stoner‐Wohlfarth model and its combination with the Néel relaxation model, are the departing theoretical settings that connect this ΔE to the anisotropy energy (E_A_) of the nanoparticle separating two consecutive magnetic configurations in a minimum energy state (magnetization parallel to easy axes): Δ*E* = *E_A_* = *K_A_* *V*_NP_. When T is sufficiently low, *E*_T_ = *K*_B_ *T* < *K_A_* *V*_NP_ holds for very small particles (small *E_A_*) in diluted conditions (negligible interactions) and, grounds the extensive use of the  *τ*_N_ to determine the value of *K*_eff_, for a set of particles with a finite size distribution:

(2) $$\tau_{N}=\tau_{0}\exp\left(\frac{E_{A}}{k_{B}T}\right)$$

where *τ*_0_ is the characteristic relaxation time, *k*_B_ is the Boltzmann constant, *T* is the temperature, and *E_A_* is the anisotropy barrier, given by *E_A_* = *K_A_* *V*_NP_.

The relationship between  *τ*_N_, and the time of the measurement technique, *τ*_m_, defines two regimes in the behavior of the assembly, namely the blocked and the superparamagnetic states, happening respectively, below and above the blocking temperature, *T*_B_ (for which *τ*_N_  = *τ*_m_). This simple model (Equation (2)), has been exploited to infer experimentally *K*_eff‐DC_, from field‐cooling zero field‐cooling (FC‐ZFC) DC measurements, noting that the ratio between τ_m_ and τ_0_, ln(*τ*_m_/*τ*_0_), for DC measurement is typically close to 25^[^ ^19^, ^20^, ^21^ ^]^:

(3) $$K_{\mathrm{eff}\;\mathrm{DC}}=\ln\left(\frac{\tau_{m}}{\tau_{0}}\right)\frac{k_{B}T_{B}}{V}\;\approx 25\frac{k_{B}T_{B}}{V}$$

However, the experimental conditions of magnetic measurement procedures strongly depart from the ideal assumption of noninteracting MNPs, which supports the validity of this approach. In fact, the conventional sample preparation for magnetometry measurements requires the tight encapsulation of moderate amounts of a powdered sample (m > 5 mg) of MNPs in a small sample holder. In this situation of close contact between MNPs, the interparticle distance, d_ij_, may be very small, in the nanometric range, where the magnetic dipolar field that each MNPs exerts on its neighbor may be intense, modifying the energy barrier and the relaxation time that each particle experiences. This enforces the need to modify the Néel model in its simple version for non‐interacting systems (Equation (2)) to account for magnetic interactions.

As demonstrated by the different and controversial models found in the literature, providing a theoretical framework for magnetic relaxation in highly concentrated systems is complex, and has not been solved since the seminal works of Dormann–Bessais–Fiorani, suggesting an increase of ΔE due to dipolar interactions,^[^ ^22^ ^]^ and the Mørup‐Tronc model, suggesting the opposite, a decrease of ΔE,^[^ ^23^ ^]^ or complementary models derived from them.^[^ ^24^, ^25^ ^]^ According to the Dormann–Bessais–Fiorani model, τ_N_ is modified through the presence of other energy terms^[^ ^26^ ^]^:

(4) $$\tau_{N}=\tau_{0}\exp\left(\frac{\Delta E^{*}}{k_{B}T}\right)$$

where the ΔE includes the contribution of *E*_A_, the energy caused by external (*E*_H_) and magnetic dipolar (*E*_D_) fields present in the system: ΔE* = *E*_A_ + *E*_H_ + *E*_D_. Therefore, although *E*_H_ can be lowered by applying extremely low fields (H < 10 Oe), the unavoidable concurrence of dipolar interaction is a persistent limitation to the use of Equation (3) as a valid formalism to obtain *K*_eff_ for concentrated systems, leading to incorrect anisotropy data^[^ ^27^ ^]^ and remains an open question in the field of MNPs.

Also, with numerical simulations, different approaches have been pursued to gain insights into the magnetic properties of ensembles of interacting single‐domain magnetic particles. In this regard, the approach of Garcia‐Otero et al.,^[^ ^28^ ^]^ in the same manner as the D‐B‐F model, considers ΔE as the sum of different energy terms:

(5) $$E=\sum_{i}E_{A}^{\left(i\right)}+\sum_{i}E_{H}^{\left(i\right)}+\frac{1}{2}\sum_{i}\sum_{i\;\neq \;j}E_{D}^{\left(i,j\right)}$$

Here, a semi‐classical approximation of µ_**i**_, the magnetostatic moment is taken as a classical vector, and the uniaxial‐type anisotropy of each i‐particle is given by:

(6) $$E_{A}^{\left(i\right)}=-KV_{i}{\left(\frac{{\vec{\mu }}_{i}\cdot \;{\vec{n}}_{i}}{\left|{\vec{\mu }}_{i}\right|}\right)}^{2}$$

where *K* is the anisotropy constant and the unit vector $\vec{n_{i}}$ denotes the easy directions; the Zeeman energy of a MNP exposed to an external magnetic field H is given by:

(7) $$E_{H}^{\left(i\right)}=-{\vec{\mu }}_{i}\cdot \;\vec{H}$$

and the magnetic dipolar interaction between two particles i and j separated by a distance d_ij_ given by:

(8) $$E_{D}^{\left(i,j\right)}=\frac{{\vec{\mu }}_{i}\cdot {\vec{\mu }}_{j}}{d_{\mathrm{ij}}^{3}}-\frac{3\left({\vec{\mu }}_{i}\cdot {\vec{d}}_{\mathrm{ij}}\right)\left({\vec{\mu }}_{j}\cdot {\vec{d}}_{\mathrm{ij}}\right)}{d_{\mathrm{ij}}^{5}}$$

This model has recently made it possible to shed light on the role of dipolar interactions from Monte Carlo simulations in systems based on MNPs,^[^ ^29^, ^30^, ^31^, ^32^, ^33^ ^]^ helping to optimize the performance of MNPs in magnetic hyperthermia processes, among others.

In addition, experimental approaches using different strategies have been essayed, trying to reduce or control MNP dipolar interactions, but reaching no consensus to date. In some cases, MNPs are either embedded and fixed within non‐magnetic matrices^[^ ^34^, ^35^, ^36^ ^]^ to determine *K*_eff_ in weakly interacting environments, or dispersed in different liquid media at known concentrations,^[^ ^37^, ^38^ ^]^ or measured in dried conditions by using non‐magnetic functional coatings as spacer systems^[^ ^39^, ^40^, ^41^, ^42^, ^43^ ^]^ to control and reduce exchange or dipolar interactions,^[^ ^44^ ^]^ and, simultaneously improve the biocompatibility of MNPs.^[^ ^45^ ^]^ More specifically, although magnetic studies performed with MNPs dispersed in liquids provide crucial insights into the Brownian and Néel relaxations in magnetic hyperthermia processes, they have limitations, like the lack of perfect colloidal stability of ferrofluids, and the formation of aggregates or precipitates, which occur to a non‐measurable extent when exposed to alternating magnetic fields. The presence of inter‐ and intra‐aggregate dipolar interaction effects, known to modify the heating efficiency of MNPs,^[^ ^46^ ^]^ introduces a source of uncertainty in the determination of *K*_eff_, given that there is no experimental control on the colloidal state of individual MNPs during the measurement procedure. This fact may be behind some of the contradictions between the observations performed using ferrofluids,^[^ ^47^ ^]^ compared to MNPs supported in solid matrices or dusty preparations. *K*_eff_ values of 18, 16, 17, and 15 kJ m^−3^ have been determined in ferrofluids (MNPs with D = 11.9 nm)^[^ ^47^ ^]^ at concentrations ≈1.24, 2.47, 4.95 and 9.89 v/v%, showing unclear trend of *K*_eff_ with concentration, where similar values, 18 and 17 kJ m^−3^, are obtained for low and high concentrations (1.24 and 4.95 v/v%). Different trends are reported in other studies using FC‐ZFC measurements to analyze lowly concentrated maghemite MNPs (D = 11.6 nm) dispersed in liquid systems (8.14, 4.65, 1.89 mg mL^−1^)^[^ ^48^ ^]^ showing a decrease in *T*_B_ as concentration increases. However, for similar maghemite MNPs of ≈11 nm,^[^ ^49^ ^]^ both trends are observed: *T*_B_ increases with interactions when in the high‐packing regime (i.e., presence of high interactions) but decreases with increasing interactions in the low‐packing regime (i.e., almost no interactions). By AC magnetometry at room *T* performed also on ferrofluids, *K*_eff_ has been obtained from the coercive field,^[^ ^50^ ^]^ although the effect of dipolar interaction cannot be explicitly computed with this method. More recently, efforts have been made to obtain precise values of *K*_eff_^[^ ^51^ ^]^ by a similar strategy, determining the blocking frequency, from AC magnetometry, for a system of very dilute MNPs with the aim of reducing the dipolar interaction, but without a precise control over it.

The discrepancy of these results is not only the result of the strategy selected to determine *T*_B_ or *K*_eff_, but also due to the fact that in none of these works magnetic interactions are explicitly decoupled from *E*_A_  or by the non‐measurable presence of aggregates within the ferrofluid samples. This problem is partly solved by determining the *E*_A_ of powdered MNPs coated with non‐magnetic shells that ensure the isolation of individual magnetic cores. In this regard, different powdered samples of MNPs have been studied using ZFC‐FC curves to compute *K*_eff_. Maghemite MNPs^[^ ^52^ ^]^ with sizes between 6.2 and 11.5 nm coated with a thick shell (17–33 nm) of SiO_2_ to avoid interactions have a *K*_eff_ of 20 and 50 kJ m^−3^ respectively. Also, magnetite MNPs using large SiO_2_ shells and different shapes (spherical, cubic, and octahedral) have been shown to present *K*_eff_ values between 10–20 kJ m^−3^.^[^ ^53^ ^]^ However, besides the efforts made up to now, to avoid the effect of dipolar interaction on the determination of *K*_eff_ by measuring MNPs with large shells, the explicit experimental determination of the magnetic dipolar interaction in terms of the interparticle distance and magnetic moment is yet to be found. Therefore, the experimental verification of the relationship between *E_D_* and d_ij_, *E_D_* ≈1/d_ij_^3^, is not only relevant to confirm this mean‐field prediction of a fundamental relationship in magnetism, analyzed by means of theoretical^[^ ^24^, ^25^ ^]^ or numerical approaches.^[^ ^28^ ^]^ Moreover, noting the high thermal insulation capacity of SiO_2_,^[^ ^54^ ^]^ optimizing the thickness of silica shells in coated MNPs, with a convenient narrow size distribution, is especially important in magnetic hyperthermia applications to ensure a compromise between maximum heat release and dipolar interaction control, when required.

In this work, an experimental strategy for the accurate determination of *K*_eff_ has been proposed to identify the energy contributions of magnetic dipolar interactions by using a model system based on iron oxide magnetic cores coated with size‐controlled silica shells acting as interspacing material. Externally applied magnetic fields were kept low to minimize the contribution of *E_H_*. Spherical iron oxide MNPs with different sizes (8, 10, and 15 nm) were obtained using coprecipitation and thermal decomposition methods, and further functionalized with SiO_2_ shells of different thicknesses, to ensure customized d_ij_, spanning from d_ij_ ≈ 10 nm to d_ij_ ≈ 45 nm to gain control of dipolar interactions at an experimental level.

Through the experimental determination of the blocking temperature with DC and AC magnetometry, the ΔE was inferred from Equation (4), for all MNP sets, showing to decrease largely with the increment of the interparticle distance, as ≈1/d_ij_^3^ in accordance with the contribution of magnetic dipole interaction. Following this experimental evidence, the Δ*E* is considered to be due to the contribution of *E*_A_ and *E*_D_. Subsequently, the fitting of the experimental data of this model system MNPs to Δ*E* = *E*_an_ + *E*_D_ (1/d_ij_^3^) has allowed us to determine *E_A_* from the asymptotic value of the energy barrier for the absent magnetic dipole interaction (d_ij_ → ∞). This asymptotic determination of *E_A_* makes it possible to decouple the anisotropy effects from the magnetic dipole interaction, ensuring a precise determination of *K*_eff_. Dipolar interaction effects were further assessed by performing Henkel Plots on the MNPs separated by different SiO_2_ shell thickness (equivalent to d_ij_), and ΔE were determined by AC dynamic measurements under low fields (1 and 10 Oe) to reduce the externally applied magnetic energy contribution (*E*_A_, *E*_D_ >> *E_H_*). With this approach, *K*_eff_ was revealed to be, 42.6 and 48.4 (8 nm iron oxide MNPs); 23.2 and 18.3 (10 nm iron oxide MNPs); and 11.7 and 11.9 (15 nm iron oxide MNPs) kJ m^−3^, close to bulk magnetite/maghemite values and showing a decrease of its value as the magnetic core size increases. These results mean it is possible to precisely determine *K*_eff_ and help to provide better predictions of the performance of MNPs in magnetic hyperthermia processes, which are crucial as tools for cancer treatments.

## 2 Results and Discussion

### 2.1 Preparation of Nanoparticle Systems

The model system is constituted by a set of MNPs with a core‐shell structure, based on iron oxide as magnetic cores produced by co‐precipitation and thermal decomposition methods, and SiO_2_ coatings with a controlled thickness following a microemulsion method, described in the Experimental Section. The co‐precipitation method was used to obtain magnetic cores of ≈8.1 ± 2.7 and 10.2 ± 3.9 nm, while the thermal decomposition method was used to produce magnetic cores of 15.3 ± 3.4 nm. Subsequently, following the microemulsion method, the magnetic cores were coated individually with a silica shell of well‐defined thickness, obtaining a core‐shell model system with a controlled center‐to‐center distance.

The samples have been denoted as MCa ‐SX, where “MC” determines the magnetic core (iron oxide magnetic core), “S” determines the non‐magnetic material (SiO_2_ shell), “a” determines the core size (8, 10, or 15 nm) and “X” the SiO_2_ thickness in nm.

MC8‐SX model systems depart from iron oxides of 8 nm as magnetic cores, with three core‐shell samples MC8‐S2, MC8‐S5, and MC8‐S10 (with X = 2, 5, and 10 nm). MC10‐SX model systems depart from iron oxides of 10 nm as magnetic cores with four core‐shell samples MC10‐S2, MC10‐S6, MC10‐S7, and MC10‐S9 (X = 2, 6, 7, and 9 nm). Finally, MC15‐SX model systems depart from iron oxides of 15 nm as magnetic cores with four core‐shell samples MC15‐S5, MC15‐S9, MC15‐S11, and MC15‐S14 (X = 5, 9, 11, and 14 nm).

The size distribution of the MNPs was determined from transmission electron microscopy (TEM) images (See Figures S1–S3, Supporting Information).

**Figure 1:**
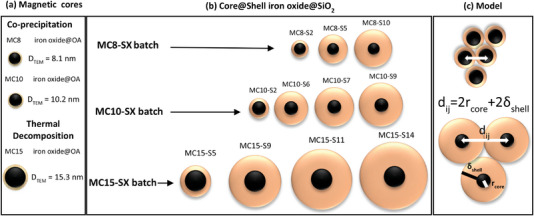
Schematic representation of the model system sets based on, a) magnetic iron oxide cores produced by co‐precipitation and thermal decomposition methods to afford different sizes (8.1, 10.2, and 15.3 nm) and, b) core‐shell MNPs with different shell thicknesses synthesized using the microemulsion method. c) With these structures it is possible to tune the distance between magnetic cores (d_ij_) by increasing the shell thickness, allowing, in turn, to control the strength of the magnetic dipole interaction.

**Figure** **1** shows a schematic representation of the different samples produced with tailored core and shell sizes as a model system to study the effect of magnetic dipolar interaction in powdered samples, were the well‐controlled silica shells act as an interspacing material.

### 2.2 Morphological and Structural Characterization

The morphological and structural properties of the synthesized MNPs were assessed using X‐ray diffraction and TEM images. **Table** **1** compiles relevant parameters thus obtained, such as the iron oxide core size (D_core_) and SiO_2_ shell thickness (D_shell_) from TEM micrographs. From the relative positions and intensities of the X‐ray diffractograms, observed in Figure S4 (Supporting Information), the crystalline phase of the synthesized MNPs, corresponding to magnetite or maghemite with an inverse spinel structure (ICSD card No. 98‐015‐8742),^[^ ^55^ ^]^ could be obtained. Moreover, it can be observed that as the thickness of SiO_2_ shell is increased, the broad peak corresponding to the presence of amorphous SiO_2_, for low scattering angles, widens, confirming the correct coating procedure.

**Table 1:** Values of core size (*D*_core_), total size (*D*_T_), and shell size (*D*_shell_) obtained from TEM micrographs by the size distribution of Figures S1–S3 (Supporting Information). The error is shown in Figures S1–S3 (Supporting Information). Blocking temperatures (*T*_B_) obtained by the ZFC‐FC curves of Figure 6. The error is <1 K for all MNPs. Magnetic saturation (*M*_S_) from hysteresis loops at 300 K. The error is <1 emu g^−1^ for all the samples. Total energy term (Δ*E*/*k*_B_) obtained from the slope of the plot ln (f/f_0_) versus 1/*T*_B_ depicted in Figure 8b and Figure S10a,b (Supporting Information) for the MC8‐SX, MC10‐SX, and MC15‐SX set, respectively. The *T*_B_ for this plot was obtained from the maximum of the *χ*″(T) curve. Effective anisotropy constant obtained by the different methods presented in this work, from *T*_B_ of the ZFC‐FC curves (*K*_eff – DC_) and from the Δ*E*/*k*_B_ term obtained by AC magnetometry (*K*_eff – AC_), considering the non‐interaction approach (Δ*E* = *E_A_*), and by the dipolar interaction correction (*K*_eff – D.Correction_) using the asymptotic limit based on Equation (15) and shown in Figure 10. The stars (*) indicate the anisotropy constant value obtained for the MNPs with the lowest effect of dipolar interactions in each batch, i.e., with the thickest SiO_2_ shell thickness

Sample core	D_core_ [nm]	Sample name	D_T_ [nm]	D_shell_ [nm]	*T*_B_ [K]	*M*_s_ [emu g^−1^]	Δ*E*/*k*_B_ [K]		*K*_eff‐DC_ [kJ m^−3^]	*K*_eff‐AC_ [kJm^−3^]		*K*_eff‐D.Correction_ [kJ m^−3^]	
							1 Oe	10 Oe		1 Oe	10 Oe	1 Oe	10 Oe
MC8	8.1	MC8‐S2	12.2	2.0	89.52	67.96	3903	3168	115.2	186.5	151.4	42.62	48.36
		MC8‐S5	18.3	5.1	59.52	69.60	1183	1485	76.6	56.5	70.9		
		MC8‐S10	27.9	9.9	51.73	66.30	1142	1205	66.6*	54.6*	57.6*		
MC10	10.2	MC10‐S2	14.7	2.3	136.91	72.38	4220	3963	90.2	104.8	98.4	23.17	18.28
		MC10‐S6	22.7	5.9	106.45	71.83	1765	1635	70.1	43.8	40.6		
		MC10‐S7	24.5	6.8	93.31	71.50	1576	1271	61.5	39.1	31.5		
		MC10‐S9	28.7	9.2	61.20	72.60	1374	1203	40.3*	34.1*	29.9*		
MC15	15.3	MC15‐S5	24.7	4.7	195.49	75.15	6334	5990	38.2	49.5	46.8	11.74	11.87
		MC15‐S9	32.3	8.5	165.09	75.73	2870	4799	32.2	22.4	37.5		
		M15‐S11	36.6	10.7	158.67	75.02	2599	2463	31.0	20.3	19.2		
		M15‐S14	43.7	14.2	154.74	75.63	2124	2045	30.2*	16.6*	16.0*		

**Figure 2:**
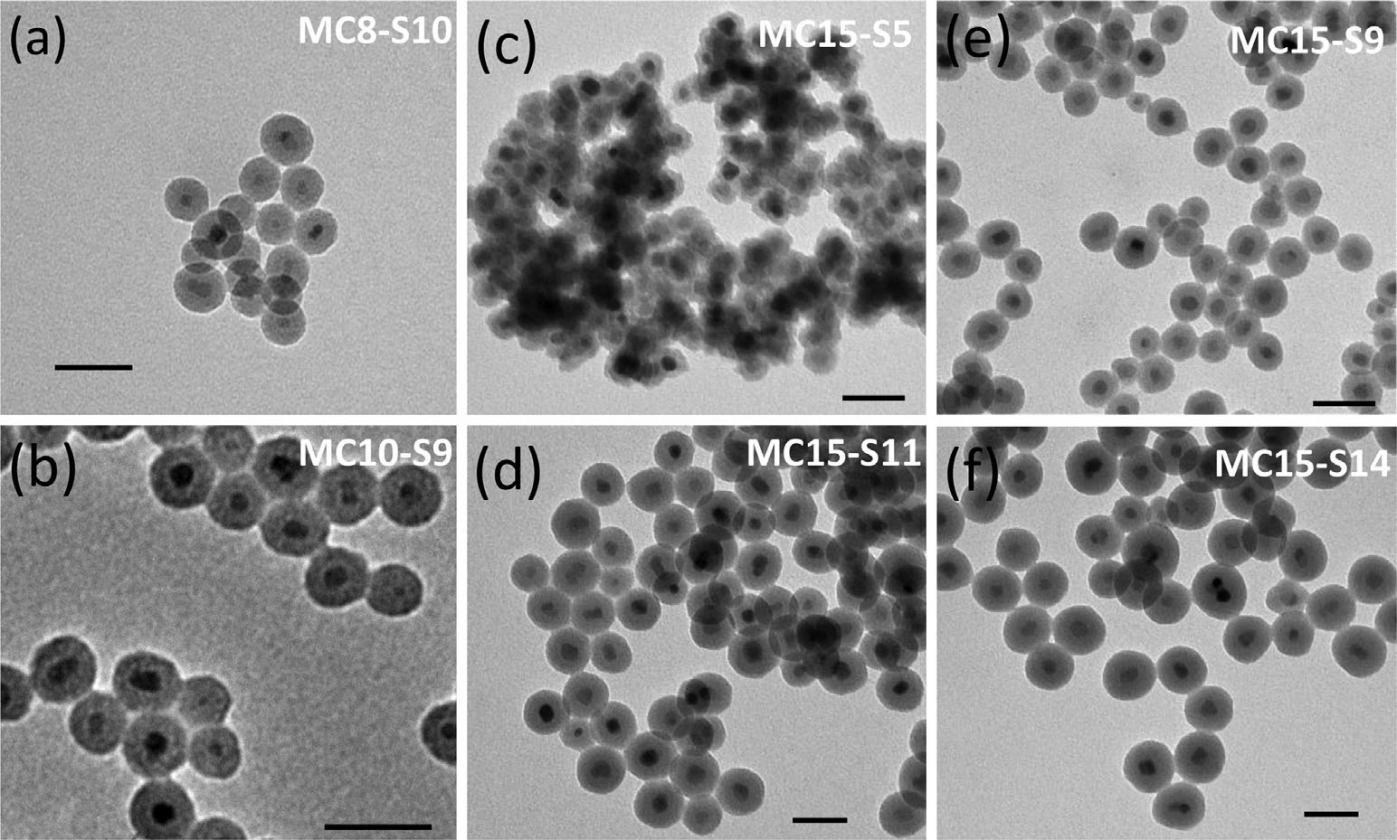
TEM micrographs of selected MNPs produced by: co‐precipitation [a) MC8‐S10 and b) MC10‐S9] and thermal decomposition [MC15‐SX MNPs: c) MC15‐S5, d) MC15‐S11, e) MC15‐S9 and f) MC15‐S14]. Scale bars: 50 nm.

The morphology and size of iron oxide MNPs, characterized by TEM micrographs are shown in **Figure** **2**. These images, show a selected set of core‐shell MNPs obtained by the co‐precipitation method (MC8‐S10, (a), and MC10‐S9 (b)) and the complete batch of MC15‐SX produced by the thermal decomposition method: MC15‐S5 (c), MC15‐S9 (d), MC15‐S11 (e) and MC15‐S14 (f), clearly reveal the homogeneous silica coating, the quasi‐spherical shape and the monodisperse size distribution of all samples.

From these data, the size and the shell thickness of all samples were determined, and Table 1 compiles the size of the uncoated magnetic cores (MC8, MC10, MC15) and their respective core‐shell iron oxide@SiO_2_ MNPs (MC8‐SX, MC10‐SX, and MC15‐SX). Due to the synthetic procedures performed, it was possible to develop MNPs with SiO_2_ shells that are sufficiently small for all MNP batches, ≈2.0 (MC8‐S2), 2.3 (MC10‐S2), and 4.7 nm (MC15‐S5), and sufficiently large, ≈9.9 (MC8‐S10), 9.2 (MC10‐S9), and 14. 2 nm (M15‐S14). This set of MNPs constitutes a model system with a controlled variation of the dipolar interactions, from strong interaction effects between close MNPs (d_ij_ < 5 nm) to weak dipolar interactions for MNPs separated by a considerable distance. Additionally, the polydispersity index (PDI) was determined from the size distribution to assess the differences in the dispersity between the different types of synthesis. PDI obtained for the magnetic cores was revealed to be similar for the MNPs synthesized by the co‐precipitation method (PDI = 0.111 for the MC8 and PDI = 0.146 for the MC10) and slightly lower for the magnetic cores synthesized by the thermal decomposition method (PDI = 0.052 for the MC15), as expected.^[^ ^56^ ^]^ In addition, PDI becomes smaller as the SiO_2_ shell size increases in all batches, from 0.092 (MC8‐S2) to 0.040 (MC8‐S10), from 0.070 (MC10‐S2) to 0.018 (MC10‐S9), and from 0. 028 (MC15‐S5) to 0.020 (MC15‐S14), in agreement with a recently reported work in which the monodispersity of spions@SiO_2_ MNPs increases with increasing SiO_2_ thickness.^[^ ^57^ ^]^

### 2.3 DC Magnetic Characterization

#### 2.3.1 Hysteresis Loops

**Figure 3:**
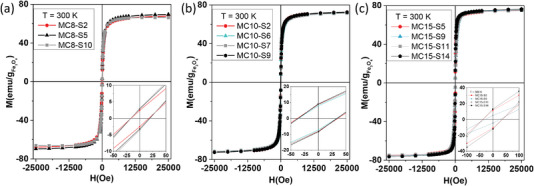
Hysteresis loops of a) MC8‐SX, b) MC10‐SX, and c) MC15‐SX MNPs at 300 K. Insets: Scale amplification in hysteresis loops. The hysteresis loops were normalized to the mass of magnetic material (iron oxide) in the total assembly.

The DC magnetic properties of MNPs were studied from magnetization curves obtained using a superconducting quantum interference device (SQUID) magnetometer between −25 kOe and +25 kOe at 300 K. **Figure** **3** depicts the hysteresis loops of the co‐precipitation (MC8‐SX (Figure 3a) and MC10‐SX (Figure 3b)) and thermal decomposition (MC15‐SX MNPs (Figure 3c)) samples. The magnetization of all MNPs was normalized to the magnetic mass present in the sample. The saturation magnetization (*M*_S_) values for all samples, compiled in Table 1, are comprised between 67–75 emu g^−1^. Batches MC8‐SX and MC10‐SX produced by co‐precipitation show *M*_S_ between 69 and 71 emu g^−1^, while batch MC15‐SX, obtained by thermal decomposition, has a slightly larger *M*_S_ with a value of ≈75 emu g^−1^, closer to the saturation corresponding to bulk magnetite (M_S_^bulk^  = 92 emu g^−1^). These differences may be attributed to the contribution of a surface dead magnetic layer (proportionally larger for smaller particles), which leads to a reduction of the magnetization of the material,^[^ ^58^ ^]^ and due to the synthetic methods used in each case, producing slight differences in the oxidation state or crystalline quality of the ensembles. In this sense, the co‐precipitation method, an easy and cost‐effective procedure using low temperatures (*T* ≈ 60 °C), cannot provide samples with a crystalline quality as high as those obtained using thermal decomposition methods, performed at higher temperatures (300 °C), which, in turn, allows to improve the magnetization of the MNPs.^[^ ^56^ ^]^

#### 2.3.2 Determination of Dipolar Interactions

One of the most widespread strategies for the study of interactions between MNPs is the Henkel plots (*δ*M), obtained from remanence studies on DC measurements.^[^ ^59^ ^]^ This technique is mainly based on the study of magnetic remanence through two curves known as isothermal remanent magnetization (IRM, or MR(H)) and DC demagnetization (DCD, or MD(H)) whose main difference, from the experimental point of view, lies in the initial saturation of the sample. While, in IRM curves, the remanence is measured in a non‐saturated state, in DCD curves the sample is initially saturated, and remanence is subsequently measured. In this work, IRM curves were obtained by applying a positive magnetic field and recording the remanence of the sample once the field was removed. The IRM curve was completed by repeating this process incrementing the field intensity until the saturation state was reached. On the other hand, to obtain the DCD curve, first, a magnetic field was applied to saturate the sample and then the remanence was measured after the application and removal of progressively increasing reverse fields.^[^ ^59^, ^60^ ^]^ IRM and DCD curves are connected by the Wohlfarth relationship^[^ ^61^ ^]^:

(9) $$M_{D}\;\left(H\right)=M_{\mathrm{RS}}-2M_{R}\left(H\right)$$

Where *M*_D_ is the DC demagnetization, *M*_RS_ is the saturation remanence, *M*_R_ is the isothermal remanence magnetization and *H* is the applied magnetic field.

From *M*_D_ and *M*_R_, it is possible to obtain the *δ*M plots by:

(10) $$\delta M\left(H\right)=M_{D}\left(H\right)-\left[M_{\mathrm{RS}}-2M_{R}\left(H\right)\right]$$

From the positivity or negativity of the dip in the *δ*M curves, it is possible to assess the presence of exchange interactions^[^ ^62^ ^]^ or dipole interactions,^[^ ^63^ ^]^ respectively. Furthermore, the absence of the dip in the *δ*M curve is characteristic of a non‐interacting MNPs system.^[^ ^64^ ^]^

**Figure 4:**
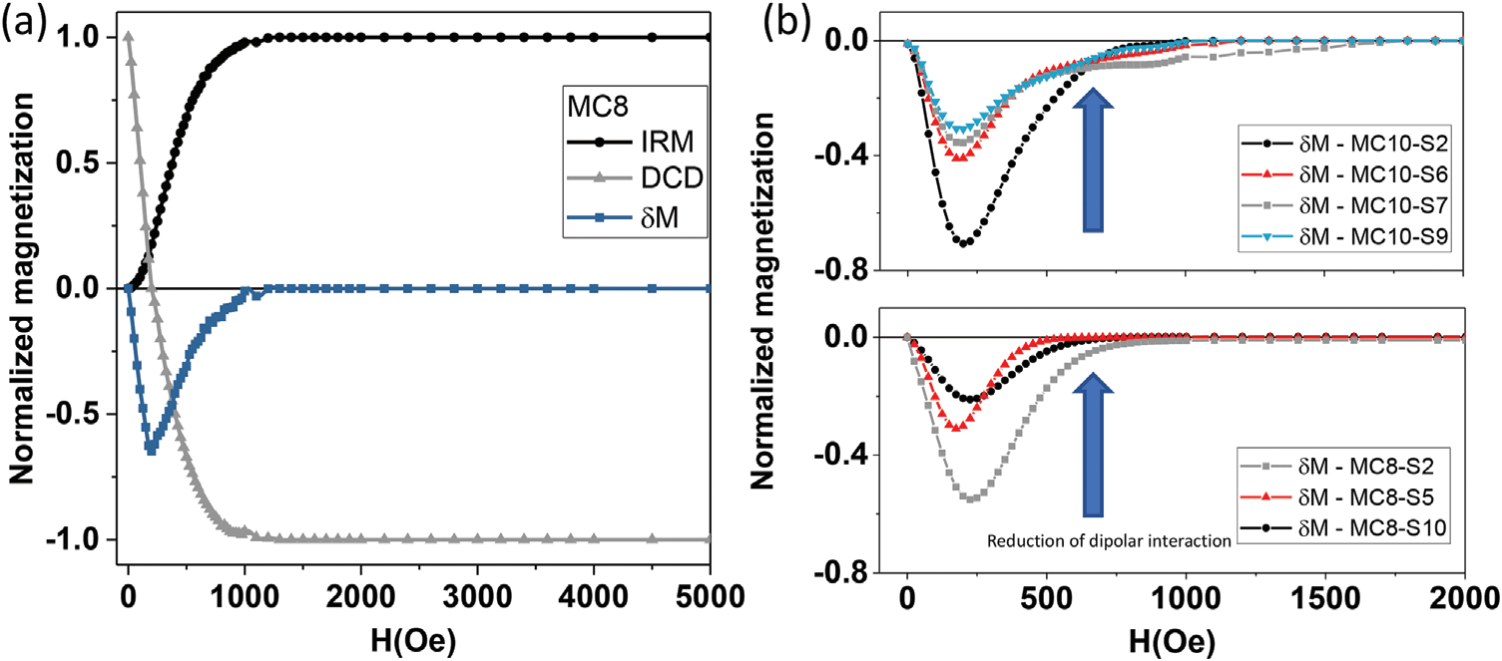
a) Normalized isothermal remanence (IRM), DC demagnetization remanence (DCD), and *δ*M curves of MC8 MNPs at 10 K. b) *δ*M curves of MC8‐SX MNPs (Bottom): MC8‐S2 (gray pattern), MC8‐S5 (red pattern) and MC8‐S10 (black pattern) and MC10‐SX MNPs (top): MC10‐S2 (black pattern), MC10‐S6 (red pattern), MC10‐S7 (gray pattern) and MC10‐S9 (blue pattern) at 10 K. The blue arrow indicates the reduction of dipole interactions with decreasing Henkel plot intensity.

**Figure 5:**
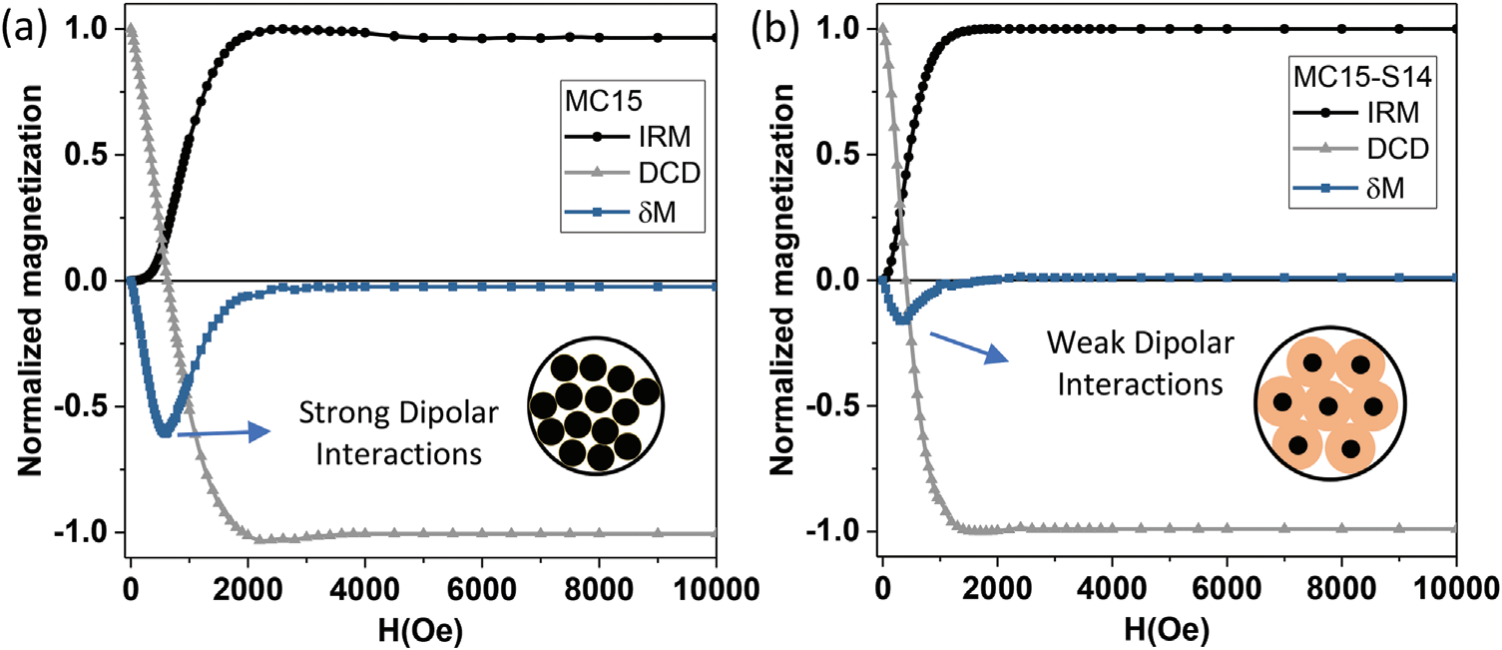
a) IRM, DCD, and *δ*M curves of MC15 MNPs (the magnetic core of MC15SX set) at 10 K and b) MC15‐S14 MNPs (MNP with largest SiO_2_ thickness of MC15‐SX set).

**Figure** **4**a shows the IRM (black points), DCD (gray points), and the *δ*M (blue points) plots of the MC8 MNPs, the magnetic core of the MC8‐SX set. The negative dip is the feature that demonstrates the existence of dipole interactions rather than exchange interactions, which can be explained by the fact that iron oxide cores were coated with a narrow layer of oleic acid (*δ* ≈ 1 nm), to prevent direct contact between the iron oxide grain and, thus, exchange interactions. Figure 4b shows the *δ*M curves of the core‐shell sets MC8‐SX MNPs (bottom): MC8‐S10 (black pattern), MC8‐S5 (red pattern), and MC8‐S2 (gray pattern) and MC10‐SX MNPs (top): (MC10‐S2 (black pattern), MC10‐S6 (red pattern), MC10‐S7 (gray pattern) and MC10‐S9 (blue pattern) performed at 10 K. From these curves it can be observed that none of the sets experience exchange interactions (positive dip). Only dipolar interaction is present, that accordingly shows to have a strength (dip depth) that decreases with the growth of the MNPs SiO_2_ thickness which is acting as a spacer. **Figure** **5** shows the IRM, DCD, and *δ*M curves of both extremes of set MC15‐SX, a) the iron oxide core of MC15 (smallest interparticle distance with OA coating of ≈1 nm), and b) MC15‐S14 MNPs (sample with greater SiO_2_ thickness, *δ* = 14 nm), revealing a strong reduction of the dip intensity for the core‐shell sample, corresponding to a drastic reduction of dipolar interaction strength.

Generally, an increment of the shell width (*δ*) provokes a reduction of the dip depth in *δ*M, which is in concordance with the fact that the magnetic dipolar interaction decreases when the interparticle distances increase (d_ij_ = 2*δ*). In all cases the reduction of dipole interactions (dip depth) correlates with large d_ij_, that is, for set MC8‐SX, the dip intensity becomes reduced to a half from −0.55 (MC8‐S2 with *δ* ≈ 2 nm) to −0.20 (MC8‐S10 with *δ* ≈ 10 nm); for set MC10‐SX, from ‐0.72 (MC10‐S2 with *δ* ≈ 2 nm) to −0.34 (MC10‐S9 with *δ* ≈ 9 nm) or, for set MC15‐SX from −0.60 (MC15 with *δ* ≈ 1 nm) to −0.15 (MC15‐S14 with *δ* ≈ 14 nm).

Moreover, to study the possible presence of exchange interactions in very small distance ranges, i.e., for the MNPs with thinner SiO_2_ thickness and even for the magnetic cores coated with OA, exchange bias field (*H*_EB_) was determined measuring hysteresis loops at *T* = 5 K under the cooling field of H_FC_ = 25 kOe (See Figure S5 (Supporting Information) for *H*_EB_ values). Almost a negligible shift of the loops was obtained in all cases (<4 Oe), and, most remarkably, no significant differences were observed between the MNPs irrespective of their shell composition (OA, or SiO_2_) or their thicknesses. It can be observed that the loop shift for the silica‐coated MNPs with the thickest silica layer (where the presence of exchange interaction is clearly ruled out), is similar to the OA‐coated magnetic cores or the silica‐coated MNPs with thin layers, where dipolar interaction may be stronger. Thus, these similarities in the shifts of the hysteresis loops reveal the marginal role of the interparticle exchange interactions in our model system, and the small values obtained can be attributed to the presence of disordered surface spins.

### 2.4 Determination of Anisotropy Constant

#### 2.4.1 Determination of Anisotropy Constant by DC magnetometry (*K*_eff‐DC_)

**Figure 6:**
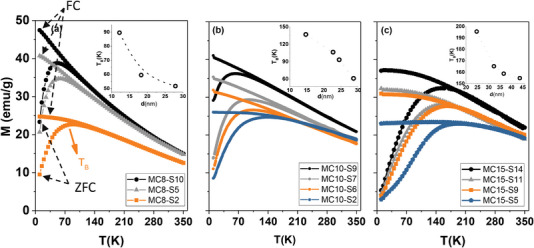
Zero‐field‐cooled (ZFC) and field‐cooled (FC) measurements of a) MC8‐SX MNPs, b) MC10‐SX, and c) MC15‐SX MNPs at 100 Oe. The blocking temperatures, observed in the insets below as a function of total nanoparticle size, i.e., the distance between magnetic cores, were obtained from the maximum of the ZFC curve and the numerical values obtained are compiled in Table 1.

To further assess the influence of the magnetic dipolar interaction, ZFC‐FC curves were determined with a SQUID magnetometer between 10 and 350 K using a magnetic field of 100 Oe. **Figure** **6** depicts the ZFC‐FC curves obtained for the different system model sets (MC8‐SX (a), MC10‐SX (b), and MC15‐SX (c) MNPs). All these curves present a maximum in temperature (*T*_Max_) in the ZFC curve, which, as in other works related with the anisotropy determination,^[^ ^53^ ^]^ has been chosen as the method to determine the *T*_B_ (although other conventions are also in operation such as the inflection point between the ZFC‐FC curves or the derivative of the difference between the FC and ZFC curves with temperature,^[^ ^65^ ^]^ being a topic of debate in the field^[^ ^66^ ^]^). *T*_B_(K) for all model systems, which represents the transition from a blocked state (*T* < *T*_B_) to a superparamagnetic state (for *T* > *T*_B_), happens below T = 200 K, ensuring superparamagnetic behavior at room temperature and are plotted in the insets of Figure 6, as a function of the silica shell or equivalently the interparticle distance. As previously observed through the Henkel plots, the increase of dipolar interactions as the interparticle distance decreases has its clear correspondence in *T*_B_. For all sets, regardless of their preparation route, small SiO_2_ shell and spacing, related to strong interactions, show a large increment in *T*_B_(K), in some cases reaching ≈200 K. On the opposite, a thick SiO_2_ shell (large spacing) is related to no or scarce interactions and very low *T*_B_(K) up to 50 K.

As expected, a reduction of *T*_B_ for non‐interacting systems^[^ ^39^ ^]^ is observed, although in the present study, depending on the core size, a unique minimum *T*_B_ is not attained for the largest SiO_2_ spacings (see insets in Figure 6). In all cases, the strong reduction of *T*_B_ with a reduction of interactions is evident, from the strongly interacting sets *T*_B‐Max_ = {89.52, 136.91, 195.49} K, to the lowest dipolar interaction state *T*_B‐Min_ = {51.73, 61.20, 154.74} K for batches MC8‐SX, MC10‐SX, and MC15SX, respectively. Moreover, it can be observed that *T*_B_ increases as the size of the magnetic core increases.

Another interesting feature obtained from the ZFC‐FC curves (Figure 6) is the signature of the interactions between MNPs that clearly appears on the FC curve shape in the irreversibility region (below the *T*_B_). As SiO_2_ thickness increases, the slope of the FC curve gradually increases and becomes steeper (weaker interactions), but as SiO_2_ thickness decreases, the FC curve tends to flatten (stronger interactions).^[^ ^67^, ^68^ ^]^ This gradual trend shows that the customization of SiO_2_ shells makes it possible to gain control on the interaction effects; more specifically, this flattening of the FC curve can be quantified in relation to the magnetization value for *T*_Max_, i.e., for *T*_B_, through the equation FC_rise_ = (*M*_plateau_  – *M*_TMAX_)/M_TMAX_ enabling the estimation of the collective or individual behavior of systems based on MNPs.^[^ ^69^ ^]^ The trends obtained, observed in Figure S10 (Supporting Information), clearly reveal a collective behavior (low FC_rise_ values) for the MNPs that have a smaller distance between magnetic cores, i.e., smaller SiO_2_ thickness, MC8‐S2, MC10‐S2, and MC15‐S5, which generally decreases as the distance increases and the interaction effects are reduced. In this sense, focusing on the general trend of each of the batches, the one with the smallest magnetic core size, MC8‐SX, presents a weaker collective behavior (higher FC_Rise_ value) while for batches MC10‐SX and MC15‐SX with larger magnetic core sizes and higher magnetization, it appears to be stronger, indicating that larger SiO_2_ thickness is needed to achieve an individual behavior of the systems.

Applying the conventional approach based on Equation (3), from the *T*_B_ obtained using the ZFC‐FC protocol, *K*_eff‐DC_ was obtained and its dependence with the interaction effects, i.e., with the increase or decrease of the distance between MNPs is observed in Figure 9 for the a) MC8‐SX, b) MC10‐SX, and c) MC15‐SX MNPs (open black symbols). A clear decrease of *K*_eff‐DC_ is observed as the interparticle spacing increases and, in this sense, *K*_eff‐DC_ can be determined in the range of reduced dipole interactions for the thickest SiO_2_ shells in each of the batches, obtaining the values of 66.6, 40.3, and 30.2 kJ m^−3^ for the MC8‐S10, MC10‐S9, MC15‐S14 MNPs. The relevant effect of the reduction of the distance between MNPs is observed when the SiO_2_ shell thickness is minimal, considering the high dipolar interaction region, whose *K*_eff ‐DC_ values were revealed to be 115.2 and 90.2 kJ m^−3^ for the MC8‐S2 and MC10‐S2 MNPs (one order of magnitude higher than that of bulk magnetite, 13.5 kJ m^−3^), and 38.2 kJ m^−3^ for the MC15‐S5 MNPs. This result clearly shows the influence of the magnetic dipolar interactions in the *K*_eff‐DC_ determination with this approach.

**Figure 7:**
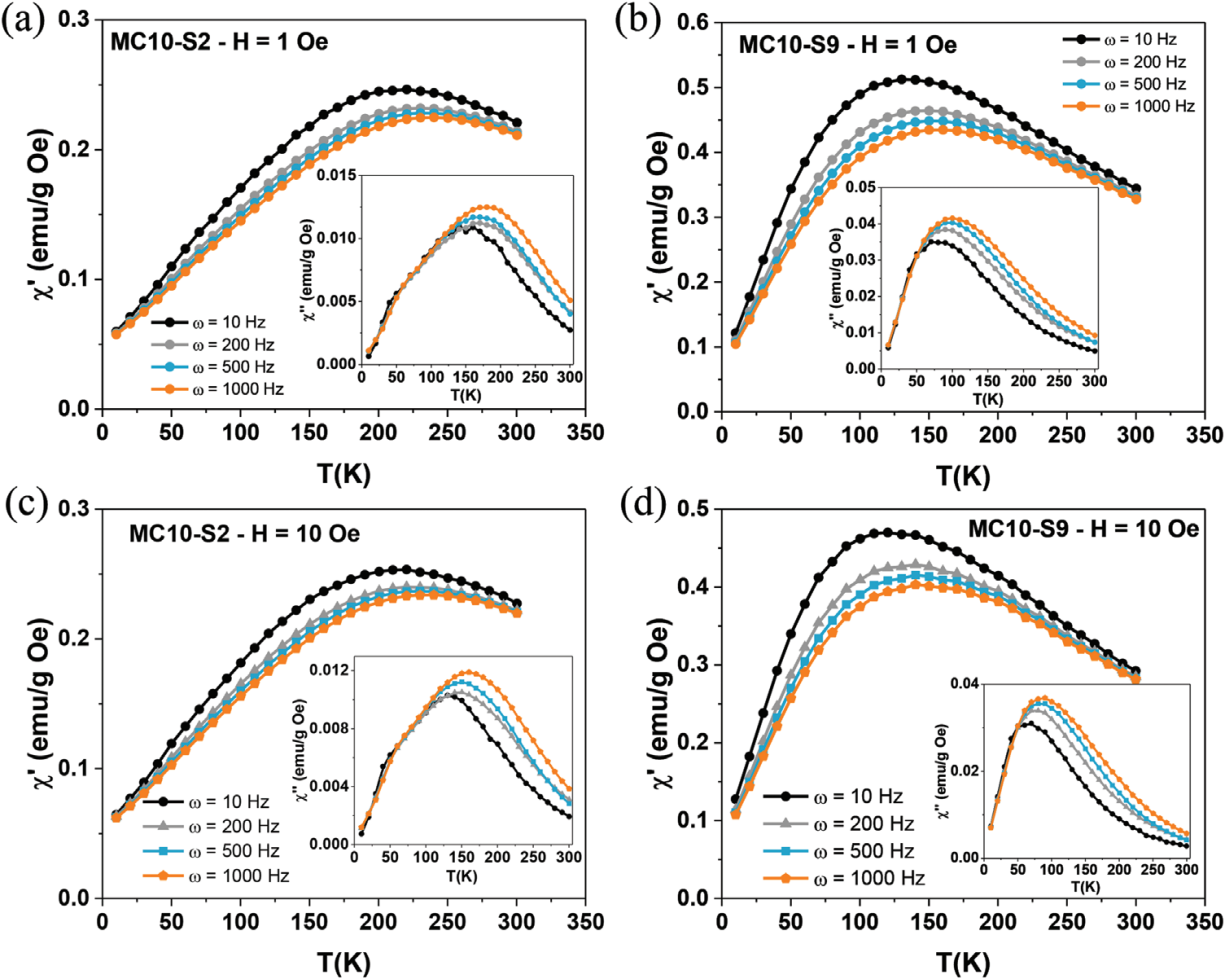
a) Temperature dependence of the real component *χ*′ (T) and *χ*″ (T) of MC10‐S2 MNPs at (a) 1 Oe and c) 10 Oe Hz and MC10‐S9 MNPs at b) 1 Oe and d) 10 Oe Hz.

#### 2.4.2 Determination of Anisotropy Constant by AC magnetometry (*K*_eff‐AC_)

From dynamic measurements, *τ*_m_ (or frequency *τ*_m_ = 1/f_m_) of each experimental technique determines the dynamic response of an ensemble of MNPs. Since the magnetic relaxation occurs through a thermally activated jump over *E*_A_, Equation (1) can be used to determine the K_eff_ by AC magnetometry, denoted as *K*_eff‐AC_, with a more precise knowledge of the time window compared to the commonly used DC experimental approach. To this end, the dynamic magnetization, real *χ*′(T) and imaginary *χ*″(T) components of the AC susceptibility of all sets (MC8‐SX, MC10‐SX, and MC15‐SX MNPs), was studied for a discrete set of frequencies f = {10, 100, 200, 1000} Hz and a range of temperatures T = [10, 300] K. **Figure** **7** shows the temperature dependence of the real part *χ*′(T) and the imaginary part *χ*″(T) of AC susceptibility at different frequencies (10 (black), 200 (gray), 500 (blue), and 1000 (orange) Hz) for MC8‐S2 MNPs at a) H = 1 and c) H = 10 Oe and MC8‐S10 MNPs at b) H = 1 and d) H = 10 Oe. These curves show a maximum for both *χ*′(T) and *χ*″(T) components, defined as *T*′_max_ and *T*″_max_. For the *χ*′(T) component, the intensity of the maximum *χ*′_max_ decreases for large frequencies, and a shift of the maximum to higher temperatures is observed. While *χ*″(T) becomes frequency independent at *T* > *T*′_max_, indicating a superparamagnetic behavior of the MNPs.^[^ ^70^, ^71^ ^]^ On the other hand, for the *χ*″(T) component, the maximum *χ*″_max_ shifts toward first higher temperatures, but its intensity increases. In Figures S6–S9 (Supporting Information), complete data of *χ*′(T) and *χ*″(T) for both fields can be found for the remaining sets (MC10‐SX, MC8‐SX, MC15‐SX). Temperature shifts enable the determination of different types of behaviors in dense MNPs systems, such as interaction regimes in concentrated ensembles of nanoparticles, interparticle spin‐glass‐like behavior due to the reduced size of the nanoparticles or the surface disorder, and collective spin‐glass‐like behavior.^[^ ^72^ ^]^ For this purpose, the parameter *ϕ*  links the shift of maximum temperatures in the susceptibility curves, *T*″_max_, and the dynamic behavior, f, through the equation *ϕ* = (Δ*T*″_max_)/(*T*″_max_ × Δlog(f)).^[^ ^71^ ^]^ Accounting for the shifts of *T*″_max_ obtained from the *χ*″(T) curve, in the frequency interval used (1000–10 Hz), *ϕ* was computed for all sets, which generally varies from non‐interaction (*ϕ* > 0.1) and considerable interaction effects (0.05 < *ϕ* < 0.1) to spin‐glass‐like behavior or high interaction effects (*ϕ* < 0.05).^[^ ^71^ ^]^ The values reached for the different batches of MNPs (in Figure S10b, Supporting Information), generally revealed a reduction of the interaction effects with increasing SiO_2_ shell, from *ϕ* > 0.05 for the MNPs with higher SiO_2_ thickness, *ϕ* ≈ 0.06, 0.08, and 0.07, (MC8‐S10, MC10‐S9, and MC15‐S14 MNPs, respectively) to *ϕ* < 0.05 for the MNPs with lower SiO_2_ shell thickness, *ϕ* ≈ 0.04, 0.04, and 0.05, (MC8‐S2, MC10‐S2, and MC15‐S5 MNPs, respectively). This makes it possible to show the reduction of interaction effects in a complementary way to the more commonly used Henkel plots.

**Figure 8:**
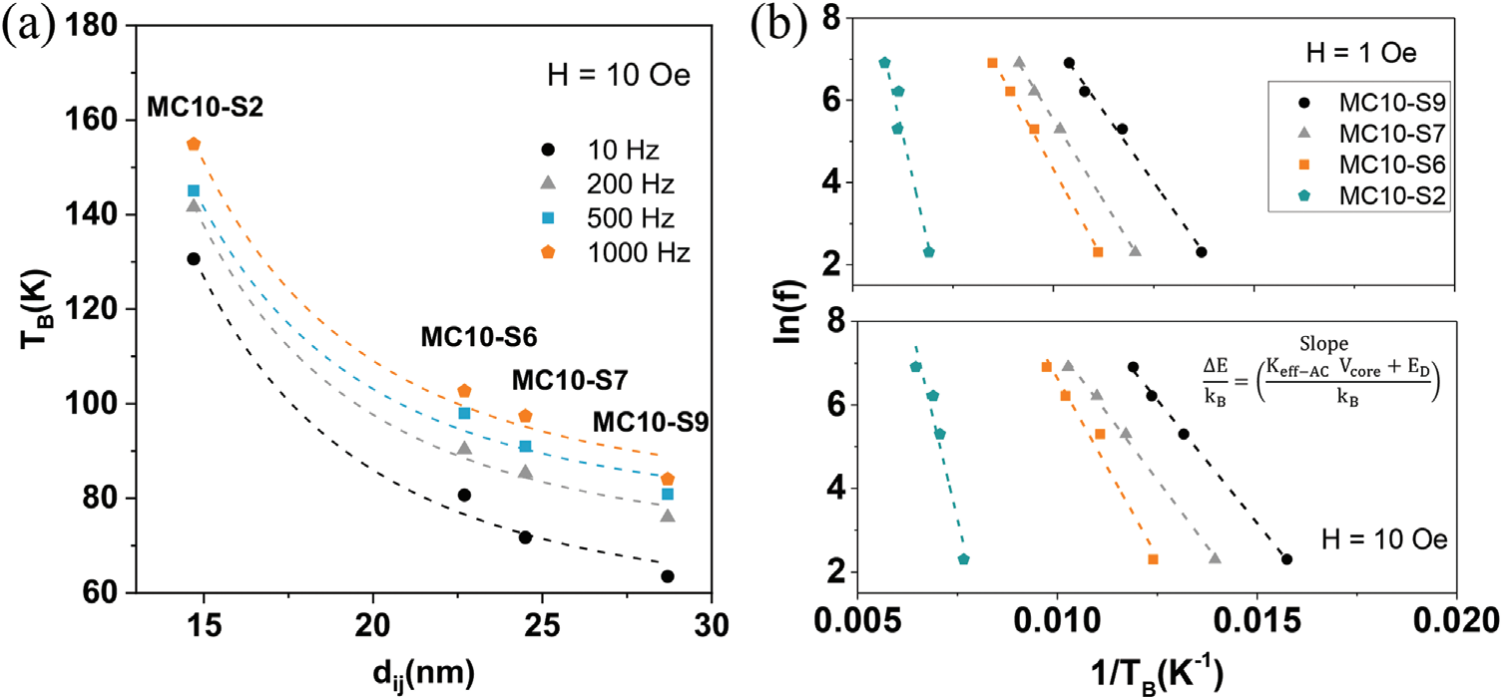
a) Dependence of T_B‐AC_ (obtained from the maximum of the *χ*″(T) curve) with an interparticle distance of the MC10‐SX batch under the application of a magnetic field of 10 Oe. b) Semilog plot of the frequency versus the inverse of the blocking temperature (obtained by the *χ*″(T) curve) for MC10‐SX MNPs at 1 and 10 Oe. Based on Equation (11) the slope of the graph makes it possible to obtain the value of the energy barrier, ΔE/k_B_, represented numerically in Table 1.

For all samples, *T*_B_ was obtained as the temperature where the maximum *χ*″_max_ occurs in the *χ*″ (T) curve, following some works reported in the field,^[^ ^71^ ^]^ although the use of *T*′_max_ as *T*_B_(K) from the curve *χ*′ (T) is also in operation.^[^ ^72^ ^]^ **Figure** **8**a shows the dependence of *T*_B‐AC_ with the center‐to‐center distance between particles d_ij_ = 2r_c_ + 2*δ* of the MC10‐SX MNPs for H_app_  =  10 Oe and all frequencies, clearly revealing the influence of dipolar interactions as observed for *T*_B‐DC_ (Figure 6). The same trend of the *T*_B_ dependence with the SiO_2_ thickness is obtained for all samples (Figure S11, Supporting Information).

Equivalent to the ZFC‐FC DC approach, where an approximation of *τ*_m_ (ln (*τ*_m_/*τ*_0_) ≈ 25) is used, by AC measurements, *K*_eff ‐AC_ can be obtained from the experimental data of the blocking temperature *T*_B‐AC_ and *τ*_N − exp_, noting that the ΔE  can be determined through the linear relationship denoted in Equation (1), which is given by the slope of the plot ln(*τ*_m_/*τ*_0_) versus 1/*T*_B_:

(11) $$\ln\frac{\tau_{N-\exp}}{\tau_{0}}=\left(\frac{\Delta E}{k_{B}}\right)\frac{1}{T_{B}}$$

In Figure 8, the experimental data of ln(f_exp_/f_0_) against 1/*T*_B_, obtained from AC *χ*″(T) measurements for the MC10‐SX set at 1 and 10 Oe (Figure S12a,b (Supporting Information) for MC8‐SX and MC15‐SX MNPs, respectively), follows a linear relationship in accordance with Equation (11). Using Equation (11), the Δ*E* *Values*, were computed and compiled in Table 1. If at this point, it is considered that the only contribution to the ΔE comes from the anisotropy term, Δ*E* = *E*_A_ (as it is commonly used in many works in the field, without considering other energy terms), *K*_eff_ can be computed noting that *E_A_* = *K_A_* *V*_NP_. In doing so, different observations emerge: for short SiO_2_ shells, the MNPs are strongly interacting, and large *K*_eff‐AC_ are obtained, for MC8‐S2, *K*_eff‐AC_ = 186.5 kJ m^−3^ (at H = 1 Oe) and 151.4 kJ m^−3^(at H = 10 Oe); for MC10‐S2, *K*_eff‐AC_ = 104.8 kJ m^−3^ (at H = 1 Oe) and 98.4 kJ m^−3^ (at H = 10 Oe); and for MC15‐S4, *K*_eff‐AC_  = 49.5 kJ m^−3^ (at H = 1 Oe) and 46.8 kJ m^−3^ (at H = 10 Oe). On the other hand, for thick SiO_2_ shells, a drastic reduction of the dipolar interaction effects (E_D_ ≈ 0) has its clear consequences on *K*_eff‐AC_, that for these cases becomes highly reduced: for MC8‐S10, *K*_eff‐AC_ = 54.6 kJ m^−3^ (at H = 1 Oe) and *K*_eff‐AC_ = 57.6 kJ m^−3^ (at H = 10 Oe); MC10‐S9, *K*_eff‐AC_ = 34.1 kJ m^−3^ (at H = 1 Oe) and 29.9 kJ m^−3^ (at H = 10 Oe); MC15‐S14, *K*_eff‐AC_ = 16.6 kJ m^−3^ (at H = 1 Oe) and 16.0 kJ m^−3^ (at H = 10 Oe) kJ m^−3^ for the three samples with the highest SiO_2_ thickness, MC8‐S10, MC10‐S9, and MC15‐S14. This highlights the relevance and distortion of the dipolar interactions in the correct *K*_eff_ determination either with DC or AC approaches.

**Figure 9:**
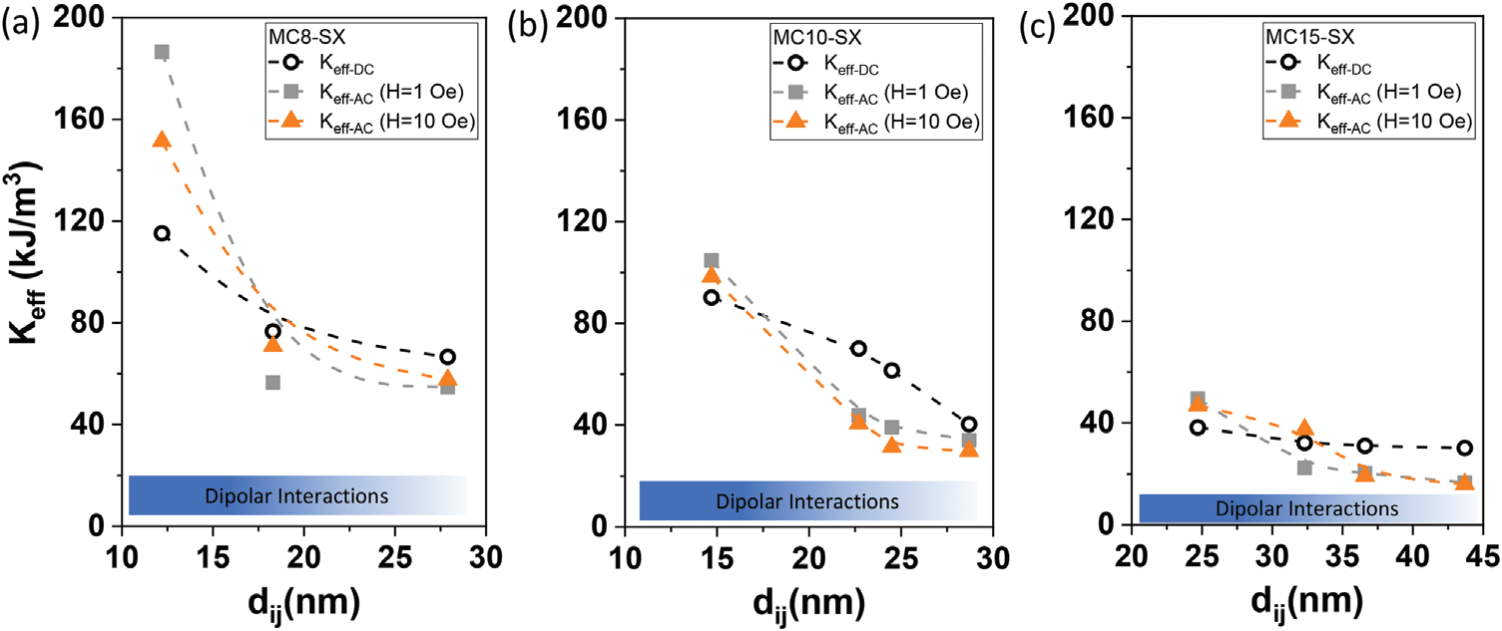
Dependence of the effective anisotropy constant on the distance between magnetic cores, that is, with increasing SiO_2_ thickness obtained by the DC (*K*_eff‐DC_, open symbols) and AC methods (*K*_eff‐AC_, close symbols) of a) MC8‐SX, b) MC10‐SX and c) MC15‐SX sets of MNPs. The dashed line represents a visual guide and the increase in the intensity of the blue color of the bar indicates the increase of the dipolar interaction strength with decreasing SiO_2_ thickness. The numerical values of *K*_eff_ are compiled in Table 1.

**Figure** **9** summarizes the values obtained using both DC (open symbols) and AC (closed symbols, orange for 1 Oe and grey for 10 Oe) methods and considering the typically used approach for non‐interacting MNPs (that takes Δ*E* = *E*_A_), as the spacing between magnetic cores decreases by decreasing the SiO_2_ thickness. These figures highlight the strong effect of dipolar interactions on the experimental determination of *K*_eff_ in dense assembled MNP systems and also, that different experimental approaches provide different *K*_eff_ which may lead to misinterpretations when designing experimental set‐ups, especially important in the field of magnetic hyperthermia.

#### 2.4.3 Determination of the Anisotropy Constant by Dipolar Interaction Correction (*K*_eff‐D.Correction_)

Although the use of sufficiently thick SiO_2_ coatings in principle is expected to minimize the error caused by applying the commonly used approach of a ΔE with no interactions (Δ*E* = *E*_A_) in the determination of *K*_eff_, accuracy is not strictly granted without a precise estimation of the intensity of magnetic dipole interactions. For this reason, this experimental approach, using a non‐interaction ΔE, can be modified following the model proposed by Garcia Otero et al., which includes two new energy terms, *E*_H_ and *E*_D_:

(12) $$\tau_{N}=\tau_{0}\exp\left(\frac{E_{A}+E_{H}+E_{D}}{k_{B}T_{B}}\right)$$

Assuming that very low H_app_ are used (H_app_ = 1 Oe; 10 Oe), *E*_H_ ≈ 0, for which only anisotropy and dipolar energies will be accounted for in a mean‐field way, and from Equation (12), the ΔE can be assumed to be Δ*E* = *K*_eff_ *V*_core_ + *E*_D_:

(13) $$\ln\frac{\tau_{N-\exp}}{\tau_{0}}=\left(\frac{K_{\mathrm{eff}}\;V_{\mathrm{core}}+E_{D}}{k_{B}}\right)\frac{1}{T_{B}}$$

Furthermore, by considering a mean‐field approach, *E*_D_ for a system of identical superparamagnetic NPs, can be roughly approached by $E_{D}=\frac{\alpha \mu^{2}}{d^{3}}$, a function of the magnetic moment of the MNPs, *µ*, and the interparticle distance, d_ij_.^[^ ^73^ ^]^ The experimental determination of Δ*E*, enables the determination of *K*_eff_ and *E*_D_ by:

(14) $$\Delta E_{\mathrm{exper}.}/k_{B}=\left(K_{\mathrm{eff}}V_{\mathrm{core}}+\frac{\alpha \mu^{2}}{d_{\mathrm{ij}}^{3}}\right)\frac{1}{k_{B}}=a+b\frac{1}{d_{\mathrm{ij}}^{3}}$$

explicitly associating the ΔE to d_ij_ and making it possible to experimentally probe the magnetic dipolar interaction for those model systems in which d_ij_ is accessible.

**Figure 10:**
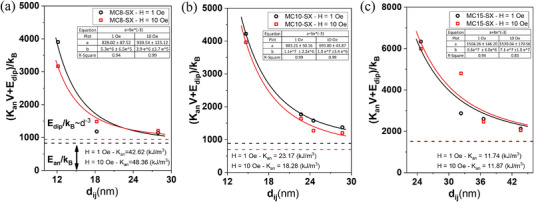
Dependence of the energy barrier formed by the sum of the anisotropy energy (*E*_A_) and the magnetic dipole interaction energy (*E*_D_) on the interparticle distance, d_ij_, and thus on the strength of the dipole interactions of a) MC8‐SX, b) MC10‐SX, and c) MC15‐SX MNPs at 1 Oe (black pattern) and 10 Oe (red pattern). The symbols represent the energy barrier values obtained by plotting ln(f/f_0_) versus 1/T_B_ based on Equation (11) and compiled in Table 1. The solid lines represent the fit of the energy barrier with the inter‐particle distance based on Equation (14). The inner box shows the values of “a” and “b” obtained in this fit, where “a” is the energy value obtained at the asymptotic limit (d_ij_ → ∞), represented by the dashed lines, which makes it possible to determine *K*_eff‐D.Correction_ by decoupling the dipole interaction effects (*E*_D_ = 0).

In **Figure** **10** the obtained experimental AC energy barriers $\frac{\Delta E_{\mathrm{exper}.}}{k_{B}}$ (symbols), obtained from Equation (11), are plotted as a function of the center‐to‐center distance between particles, d_ij_, for system models [Figure 10a MC8‐SX, Figure 10b MC10‐SX and Figure 10c MC15‐SX MNPs at H = 1 Oe (black pattern) and H = 10 Oe (red pattern)]. By fitting the energy barrier data to Equation (14), a clear d_ij_^−3^ dependence is obtained, thus experimentally confirming the contribution of a mean magnetic dipolar effect which makes it possible to decouple the interaction effect to obtain accurate *K*_eff_ values. Therefore, these results provide an experimental insight into the dependence of the dipolar energy term on the distance between MNPs, confirming the mean‐field model of energy barrier described by Garcia Otero et al., which is crucial to accurately account for magnetic dipole interactions in the concentrated ensemble of MNPs.

Accordingly, *K*_eff_ has been determined from data in Figure 10 as the asymptotic value of Equation (14) as a more precise way to obtain *K*_eff‐D.Correction_:

(15) $$\begin{array}{c} K_{\mathrm{eff}-D.\mathrm{Correction}}=\lim_{d_{\mathrm{ij}\to \infty }}\left(K_{\mathrm{eff}}\;V_{\mathrm{core}}+\frac{{\alpha \mu }^{2}}{d_{\mathrm{ij}}^{3}}\right)\frac{1}{k_{B}}=\lim_{d_{\mathrm{ij}\to \infty }}\left(a+b\frac{1}{d_{\mathrm{ij}}^{3}}\right) \end{array}$$

where d_ij_ → ∞ and the magnetic dipolar interactions become negligible.

Therefore, *K*_eff‐D.Correction_ computed as *K*_eff_ = ak_B_ for the different MNPs: for set MC8‐SX, *K*_eff‐D.Correction_ = 42.62 kJ m^−3^ (at H = 1 Oe) and, 48.36 kJ m^−3^ (at H = 10 Oe); for set MC10‐SX, *K*_eff‐D.Correction_ = 23.17 kJ m^−3^ (at H = 1 Oe) and, 18.28 kJ m^−3^ (at H = 10 Oe); and for set MC15‐SX, *K*_eff‐D.Correction_ = 11.74 kJ m^−3^ (at H = 1 Oe) and 11.87 kJ m^−3^ (at H = 10 Oe).

**Figure 11:**
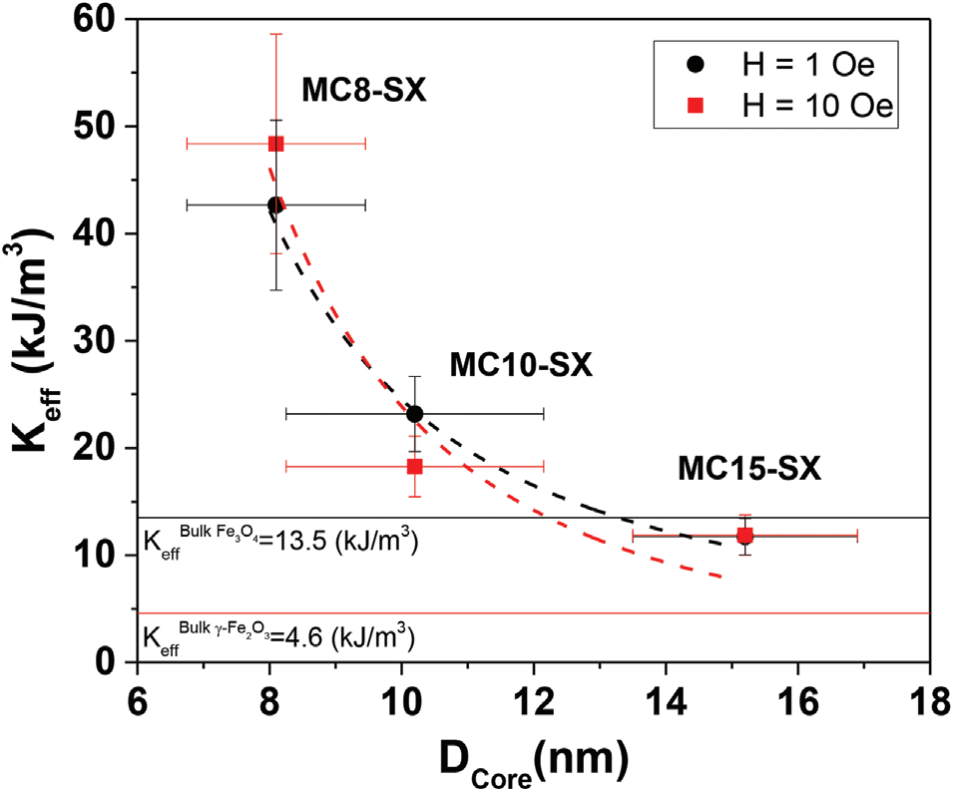
Effective anisotropy constant as a function of core size at 1 Oe (black pattern) and 10 Oe (red pattern) obtained from the dipolar interaction correction (*K*_eff‐D.Correction_). The horizontal and vertical bars represent the errors of the core size, obtained by the size distribution of the MNPs, and of the *K*_eff‐D.Correction_ was obtained based on the errors of the “a” term of the fit and of the particle volume. The solid and dashed lines represent the values of the magnetite (black) and maghemite (red) anisotropy constants of the bulk material and a visual guide to observe the decrease of *K*_eff‐D.Correction_ with particle size.

All *K*_eff‐D.Correction_ data are compiled in Table 1 and plotted in **Figure** **11** as a function of the magnetic core (8.1, 10.2, and 15.3 nm). For both H_app_, K_eff_ becomes larger for small particles, while for larger sizes, tends to the value of bulk magnetite (13.5 kJ m^−3^) and bulk maghemite (4.6 kJ m^−3^), as shown in Figure 11. This behavior is in agreement with the scaling law relationship of *K*_eff_, *K*_eff_ = *K*_b_ + 6K_s_/D,^[^ ^74^ ^]^ that at nanoscale accounts for the bulk material anisotropy, *K*_b_ and surface anisotropy, *K*_S_ surface effects, depending on the particle size. In recent studies,^[^ ^75^ ^]^ further modifications of this general relationship include additional terms accounting for the effect of a surface layer of disordered spins with thickness, d.

However, although the use of different syntheses (co‐precipitation and thermal decomposition) allows us to test the validity of the method under different conditions, possible variations in crystalline quality and stoichiometry may lead to a variation in the bulk anisotropy constant in the different cores. This fact is more noteworthy as the surface anisotropy effect is larger, that is, as the nanoparticle size decreases. Thus, although the *K*_eff_ values clearly decrease with increasing magnetic core size, no scaling law fitting has been established (Note that the dashed lines in Figure 11 represent visual guiding). On the other hand, since different iron oxide phases could coexist (mainly magnetite and maghemite) within the same nanoparticle, for example, through oxidation of the outermost surface, both bulk anisotropy constants have been selected as reference, 13.5 kJ m^−3^ for magnetite (black line in Figure 11) and 4.6 kJ m^−3^ for maghemite (red line in Figure 11). Even though OA and SiO_2_ coatings can prevent such oxidation compared to bare cores.^[^ ^76^ ^]^ Moreover, *K*_eff‐D.Correction_, so obtained for all experimental sets, is lower than the one obtained from AC magnetometry (*K*_eff‐AC_) in the non‐interacting regime when using a thick SiO_2_ shell, showing that commonly used approaches, that take large coating shells as spacers, are generally not enough to strictly avoid magnetic dipole interactions. Therefore, the present method, taking *K*_eff_ as the mathematical limit of infinite spacing, for a complete set of samples, precisely determines the anisotropy uncoupled from the magnetic dipole contribution.

## 3 Conclusion

In summary, a dipolar interaction correction for the anisotropy constant determination was carried out experimentally employing a model system of MNPs with different size cores (8.1, 10.2, and 15.3 nm) and SiO_2_ coating shells of different widths. The coating of magnetic cores with silica layers of different thicknesses has enabled the controlled reduction of interaction effects, making it possible to distinguish their contribution in the determination of the magnetic properties of MNPs. In all the developed model systems, the progressive reduction of the dipolar interactions with increasing interparticle distance was confirmed through the decrease of the intensity of the Henkel Plots dips. In the first approach, the anisotropy constant was determined from the blocking temperature obtained from DC and AC magnetometry considering the energy barrier caused only by anisotropy energy as it is typically employed for the determination of *K*_eff_ in nanoparticles with thick SiO_2_ shells. Correspondingly, values denoted as *K*_eff‐DC_ and *K*_eff‐AC_ were obtained for the MNPs with the thickest SiO_2_ shell obtaining values of 66.6, 40.2, and 30.2 kJ m^−3^ and 57.6, 29.9, and 16.9 kJ m^−3^, for the 8.1, 10.2 and 15.3 nm magnetic cores, respectively. Furthermore, the effects of strong magnetic dipole interaction were revealed by reducing the SiO_2_ thickness: small shells produce large values of *K*_eff_ due to the large contribution of magnetic dipole interaction energy. This fact highlighted the need to decouple the dipolar interaction energy from anisotropy energy, to determine precisely *K*_eff_. Therefore, with AC measurements at extremely reduced fields, to minimize the Zeeman contribution, by adding a mean field magnetic dipole interaction energy term (*E*_D_), its relationship with the interparticle distance (d_ij_) was experimentally proven and could be decoupled from the energy barrier for the precise determination of the anisotropy constant. *K*_eff_ values of 42.62, 23.17, and 11.74 kJ m^−3^ for MNPs with sizes of 8.1, 10.2, and 15.3 nm, respectively, decoupled from the contribution of magnetic dipole interaction were obtained and revealed to decrease with the nanoparticle size.

## 4 Experimental Section

### Materials

Chemicals obtained from Sigma–Aldrich used for this study were, iron(III) chloride hexahydrate (FeCl_3_·6H_2_O, 99%), iron(II) sulfate heptahydrate (FeSO_4_·7H_2_O, 99%), iron(III) acetylacetonate (Fe(C_5_H_7_O_2_)_3_, 99.9%), 1,2‐hexadecanediol (C_16_H_34_O_2_, 90%), benzyl ether (C_14_H_14_O, 98%), hydrochloric acid (HCl, 37%), ammonium hydroxide (NH_4_OH, 28%), oleic acid (OA, C_18_H_34_O_2_, 90%) oleylamine (C_18_H_37_N, 70–80%), Igepal CO‐520 (pure), tetraethyl orthosilicate (TEOS, 99%), cyclohexane (C_6_H_12_, ≥99.5%), ethanol (C_2_H_6_O, absolute) and isopropyl alcohol (IPA, C_3_H_8_O, ≥99.7%) and biological degree water, obtained from Corning, was used in all procedures.

### Synthesis of Iron Oxide Nanoparticles (MC8‐SX – Co‐precipitation Method)

MNPs with a core size of 8 nm (MC8‐SX batch) were obtained by a co‐precipitation method, following the procedure described by Vargas‐Osorio et al.^[^ ^7^ ^]^ In a typical synthesis, FeCl_3_·6H_2_O (90 mmol) and FeSO_4_·7H_2_O (60 mmol) were dissolved in 200 mL of 0.01 m HCl aqueous solution and mechanically stirred. The mixture was heated up to 60 °C, then NH_4_OH (430 mmol) and oleic acid (14.2 mmol) were added, and the reaction was carried out for 1 h. After that, the sample was transferred to a beaker and placed on a hot plate at 200 °C to enable precipitation. The precipitate containing iron oxide@OA MNPs was retained with a magnet and the supernatant was removed. The iron oxide@OA MNPs were washed three times with deionized water. Finally, the MNPs were redispersed in cyclohexane, and the remaining water was completely removed from the organic phase using a decantation funnel.

### Synthesis of Iron Oxide Nanoparticles (MC10‐SX – Co‐precipitation Method)

MNPs with a core size of 10 nm coated with oleic acid were obtained by a co‐precipitation procedure, following Massart's method^[^ ^77^ ^]^ with some modifications. In a typical synthesis, FeCl_3_·6H_2_O (90 mmol) and FeSO_4_·7H_2_O (60 mmol) were dissolved in 200 mL HCl 0.01 m under mechanical stirring at 300 rpm and 60 °C. After 15 min, 60 mL NH_4_OH (430 mmol) and oleic acid (14.2 mmol) were added, and the reaction was carried out for 1 h. The mixture color turned from brown to black. After that, the sample was transferred to a beaker and placed on a hot plate at 100 °C until flocculation occurred. The precipitate containing the MNPs was retained with a magnet and the supernatant was removed. The resulting MNPs were washed three times with deionized water and once with ethanol. Finally, the MNPs were redispersed in cyclohexane.

### Synthesis of Iron Oxide Nanoparticles (MC15‐SX – Thermal Decomposition Method)

MNPs with a core size of 15 nm coated with oleic acid were obtained by a thermal decomposition procedure. Briefly, iron acetylacetonate (12 mmol), 1,2‐hexadecanediol (48 mmol), oleic acid (96 mmol), and oleylamine (24 mmol) were dissolved in benzyl ether (120 mL) at 50 °C for 20 min with mechanical stirring at 400 rpm under N_2_ atmosphere. The mixture was heated up to 200 °C under reflux and without stirring. After 30 min, the temperature was raised to 300 °C and the reaction was left for 2 h. Once the mixture was cooled down to room temperature, the MNPs were magnetically isolated using a magnet and were washed 6 times with a mixture 1:1 of ethanol:acetone. The MNPs were redispersed in cyclohexane.

### Synthesis of Silica Coating

The coating with a SiO_2_ shell of the previously obtained iron oxide@OA MNPs was performed by using a microemulsion method, following the technique reported by Moldes et al.^[^ ^78^ ^]^ with some modifications. Briefly, iron oxide@OA MNPs dispersed cyclohexane was added to a mixture of Igepal CO‐520 and cyclohexane. The mixture was stirred at room temperature under mechanical stirring at 350 rpm for 30 min. Then, NH_4_OH and TEOS were incorporated, and the mixture was covered with aluminium foil and stirred for 16 h at room temperature. The obtained MNPs were washed 4 times, using the following solvents in the presented order: 2‐propanol, a mixture 1:1 of 2‐propanol:ethanol, ethanol, and a mixture 1:1 of ethanol:H_2_O. For each wash, the MNPs were retained with a magnet and the supernatant was removed. Finally, the MNPs were washed twice with H_2_O and centrifuged at 9000 rpm for 10 min. The MNPs were redispersed in H_2_O. The amount of NH_4_OH and TEOS in each synthesis in order to obtain different SiO_2_ layer thicknesses is shown in **Table** **2**.

**Table 2:** Amount of NH_4_OH and TEOS to obtain different SiO_2_ layer thickness in M_8_Si_X_, M_10_Si_X_ and M_15_Si_X_ batches

Magnetic core	Sample	NH_4_OH [mmol]	TEOS [mmol]	SiO_2_ layer [nm]
MC8	MC8‐S2	15	10.7	2.0
	MC8‐S5	27	18.8	5.1
	MC8‐S10	19	13.4	9.9
MC10	MC10‐S2	5	3.6	2.3
	MC10‐S6	10	6.3	5.9
	MC10‐S7	15	5.4	6.8
	MC10‐S9	15	10.7	9.2
MC15	MC15‐S5	0.35	0.22	4.7
	MC15‐S9	0.71	0.45	8.5
	MC15‐S11	1.13	0.72	10.7
	MC15‐S14	2.03	1.07	14.2

### Physicochemical, Magnetic Characterization of MNPs and Statistics

The characterization of the crystalline phases of the materials was performed by X‐ray diffraction on powder samples with a Philips PW1710 diffractometer (Cu K_a_ radiation source, l = 1.54186 Å). Measurements were collected in the 2*θ* angle range between 10° and 80° with steps of 0.02° and 10 s per step. From the diffractograms, the crystalline phase of all MNPs was determined on the basis of the Inorganic Crystal Structure Database. The morphology of the materials was characterized by transmission electron microscopy (TEM) micrographs using a JEOL JEM‐1011 microscope (100 kV). From these images the size distribution of the MNPs was determined using ImageJ software, fitting the values to a Distribution Fit using OriginPro 2016 software. For the determination of the MNP diameters, n > 100 particles were used. Iron content in the nanocomposite samples was determined by flame atomic absorption spectroscopy in a Perkin Elmer 3110 Atomic Absorption Spectrometer. DC magnetization curves of dried samples were measured using a Quantum Design SQUID magnetometer (Quantum Design, Darmstadt, Germany). Zero‐field‐cooled (ZFC) and field‐cooled (FC) curves were taken at an applied magnetic field of 100 Oe from 10 to 350 K, using a sweeping rate of 2.5 K min^−1^. In the ZFC protocol, the sample was initially cooled without the application of a magnetic field until a temperature of 10 K was reached. At this point, a magnetic field of 100 Oe was applied and the magnetization recorded as the temperature increased up to 350 K. The sample was then cooled again to 10 K under the application of a 100 Oe magnetic field and, once the temperature was reached, the magnetization was recorded again until 350 K, and the FC curve was obtained. The hysteresis loops were measured with an applied magnetic field between −25 KOe and 25 KOe at 300 K. The isothermal remanent magnetization (IRM) and the direct current DCD were measured at T = 10 K following thermal demagnetization. From these curves, the Henkel plots were computed. For the magnetic measurements, a considerable amount (at least 5 mg) of the nanocomposite was used and placed in a sample holder in a powdered state. AC magnetization curves of dried samples were measured using a Quantum Design Physical Property Measurement System in a temperature range of 10–300 K with steps of 10 K, an excitation field of 1 and 10 Oe, and driving frequencies varying from 10 to 1 KHz.

Figures 3, 4, 5, 6, 7, 8, 9, 10, 11 and all figures in the Supporting Information were obtained using Origin 2016 software. The size distribution data were given as (average ± standard deviation) for particle sizes. The errors obtained in the different fits were obtained by means of the fits through the Origin 2016 software (This is the case in Figure 8b and 10; S11, Supporting Information). The error of the *K*_eff‐D.Correction_ was determined by the indirect measurement method from the previously obtained errors of volume and the parameter “a”.

## Conflict of Interest

The authors declare no conflict of interest.

## Supporting information

Supporting Information

## Data Availability

The data that support the findings of this study are available from the corresponding author upon reasonable request.
