# Supplementary material for: Role of Dipolar Interactions on the Determination of the Effective Magnetic Anisotropy in Iron Oxide Nanoparticles
Source: Adv Sci (Weinh). 2022 Dec 12;10(5):2203397. doi: 10.1002/advs.202203397 (PMC9929252; doi:10.1002/advs.202203397)
Supplement: Supplementary file 1 — Supporting Information [file ADVS-10-2203397-s001.pdf]

ADVANCED SCIENCE
Open Access

Supporting Information

for *Adv. Sci.*, DOI 10.1002/advs.202203397

Role of Dipolar Interactions on the Determination of the Effective Magnetic Anisotropy in Iron Oxide Nanoparticles

Pelayo García-Acevedo, Manuel A. González-Gómez, Ángela Arnosa-Prieto, Lisandra de Castro-Alves, Yolanda Piñeiro* and José Rivas*

Supporting Information

Role of Dipolar Interactions on the Determination of the Effective Magnetic Anisotropy in Iron Oxide Nanoparticles

Pelayo García Acevedo, Manuel A. González Gómez, Ángela Arnosa Prieto, Lisandra de Castro Alves, Yolanda Piñeiro* and José Rivas*

I. Size Distribution

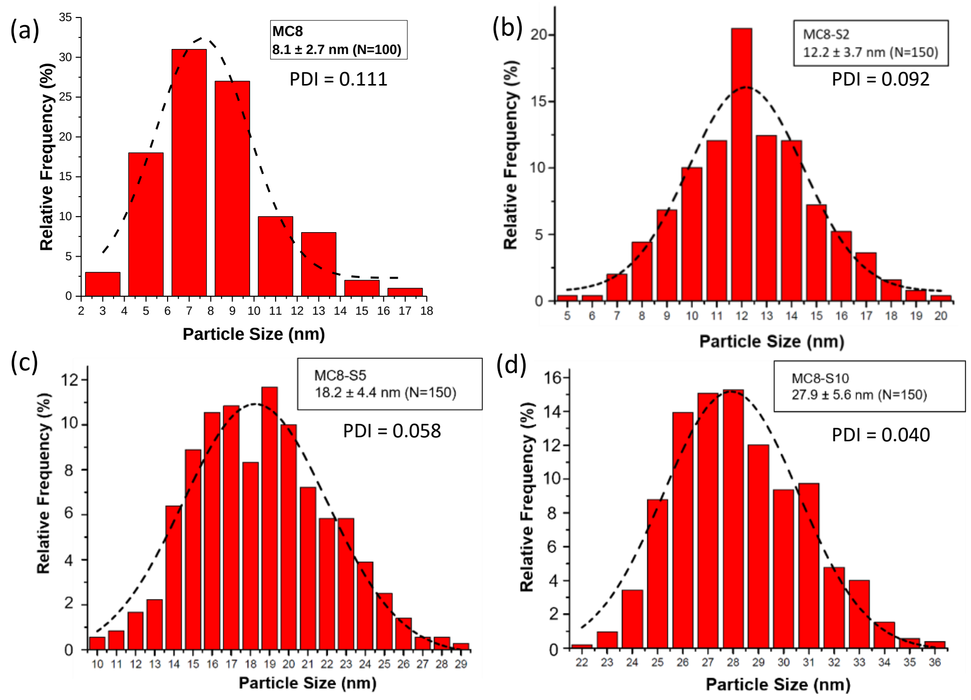

Figure S1. Size distribution histograms obtained by TEM micrographs of the MC8-SX batch: **(a)** MC8, **(b)** MC8-S2, **(c)** MC8-S5 and **(d)** MC8-S10. Size distribution was performed using Image J software.

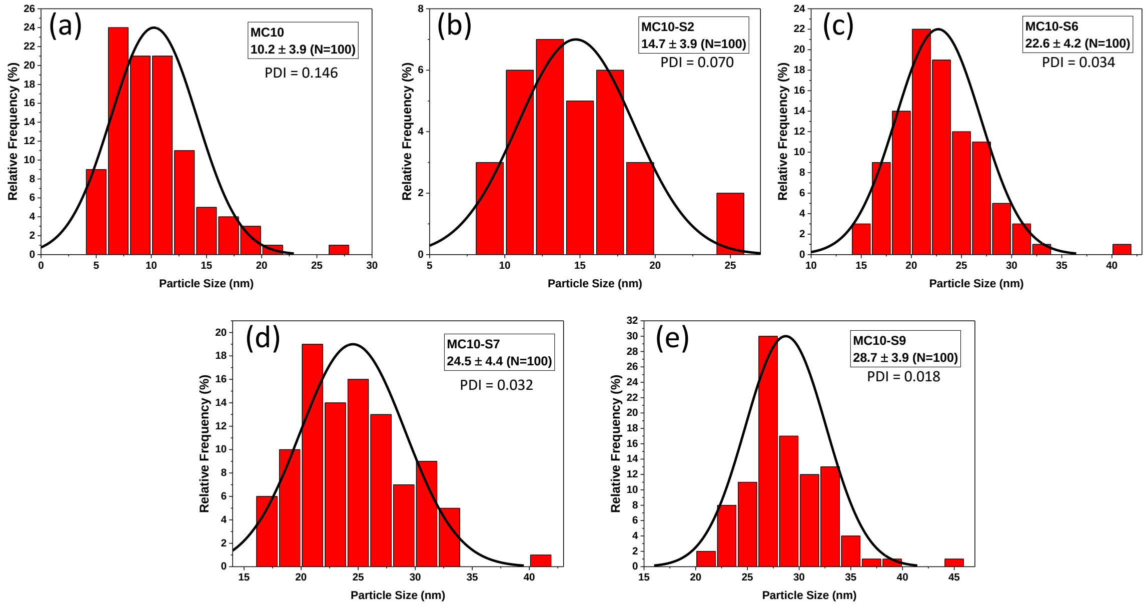

Figure S2. Size distribution histograms obtained by TEM micrographs of the M10-SX batch: **(a)** MC10, **(b)** MC10-S2, **(c)** MC10-S6, **(d)** MC10-S7 and **(e)** MC10-S9. Size distribution was performed using Image J software.

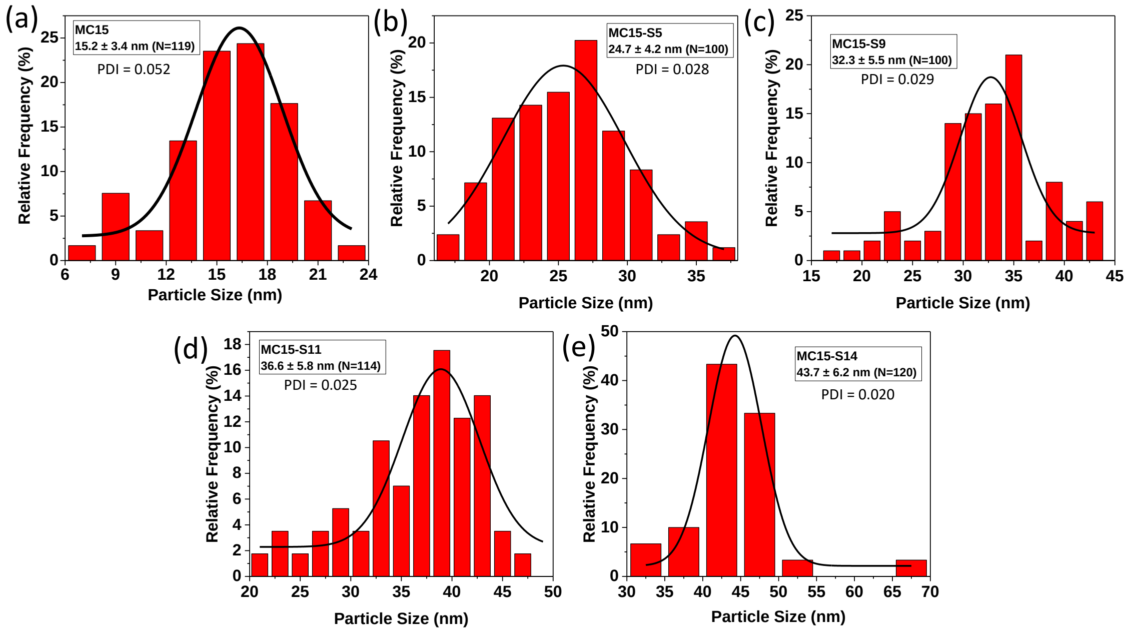

Figure S3. Size distribution histograms obtained by TEM micrographs of the M15-SX batch: **(a)** MC15, **(b)** MC15-S5, **(c)** MC15-S9, **(d)** MC15-S11 and **(e)** MC15-S14. Size distribution was performed using Image J software.

II. XRD characterization

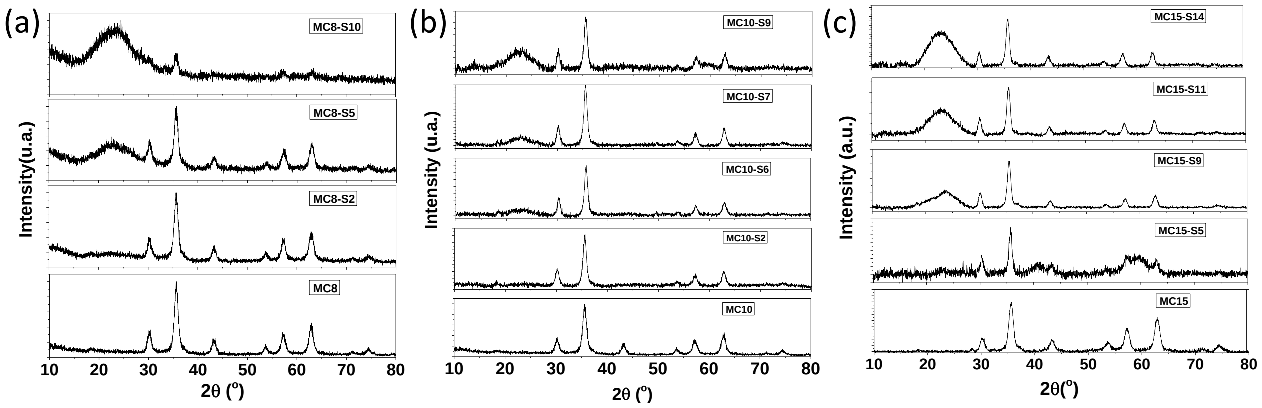

Figure S4. XRD pattern of **(a)** MC8-SX, **(b)** MC10-SX and **(c)** MC15-SX MNPs from the magnetic core without SiO_2 shell (top) to the thicker SiO_2 shell.

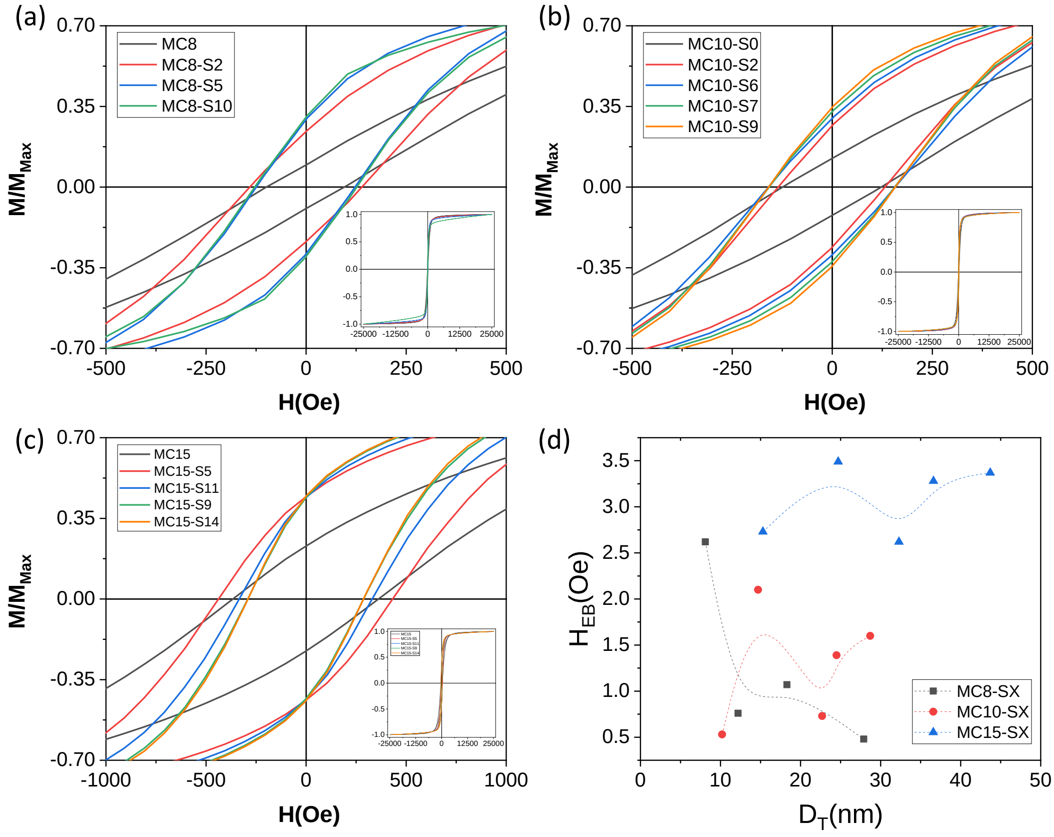

Figure S5. Hysteresis loops of **(a)** MC8-SX, **(b)** MC10-SX and **(c)** MC15-SX MNPs at 5 K cooled with H_{FC} = 10 kOe in the range of −25 kOe and 25 kOe. For clarity only the range between −0.5 kOe and 0.5 kOe is shown for the MC8-SX and MC10-SX sets and −1 kOe and 1 kOe for the MC15-SX set. Insets: Complete hysteresis loops. **(d)** H_{EB} values obtained for the different MNPs as a function of total nanoparticle size for the MC8-SX (gray), MC10-SX (red) and MC15-SX (blue) sets of MNPs. The dotted line represents visual guidance.

III. AC Magnetic Characterization

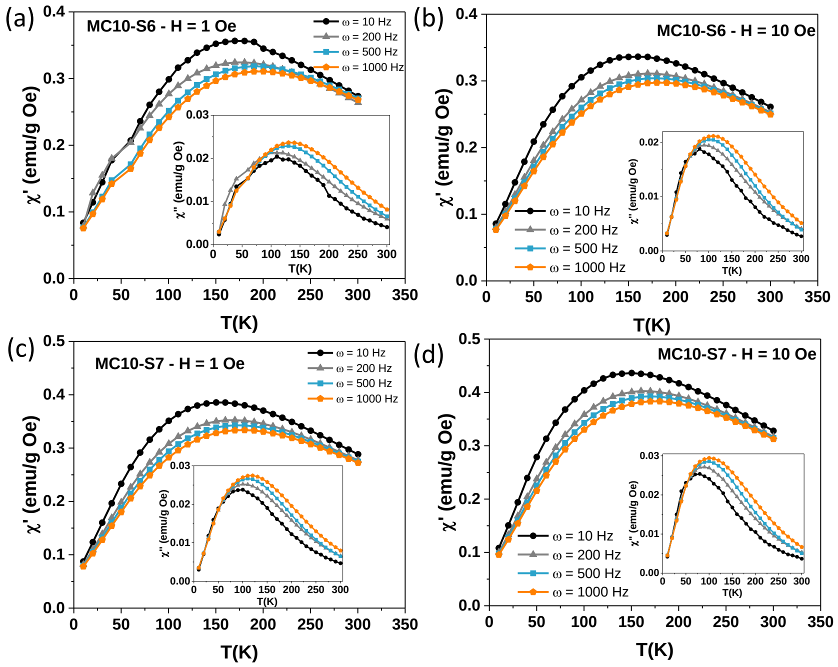

Figure S6. Temperature dependence of the real component χ'(T) and χ''(T) (inset) of MC10-S6 MNPs at **(a)** 1 Oe and **(b)** 10 Oe and MC10-S7 MNPs at **(c)** 1 Oe and **(d)** 10 Oe.

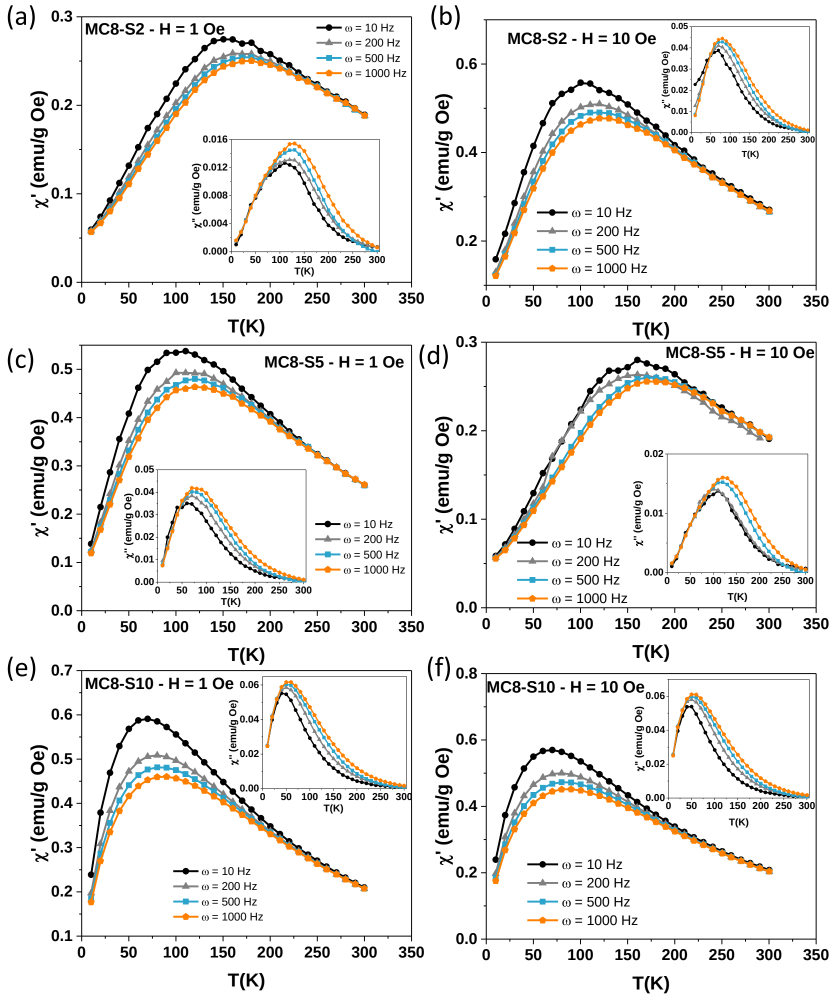

Figure S7. Temperature dependence of the real component χ’(T) and χ’’(T) (inset) of MC8-S2 MNPs at **(a)** 1 Oe and **(b)** 10 Oe; MC8-S6 MNPs at **(c)** 1 Oe and **(d)** 10 Oe; and MC8-S10 MNPs at **(e)** 1 Oe and **(f)** 10 Oe.

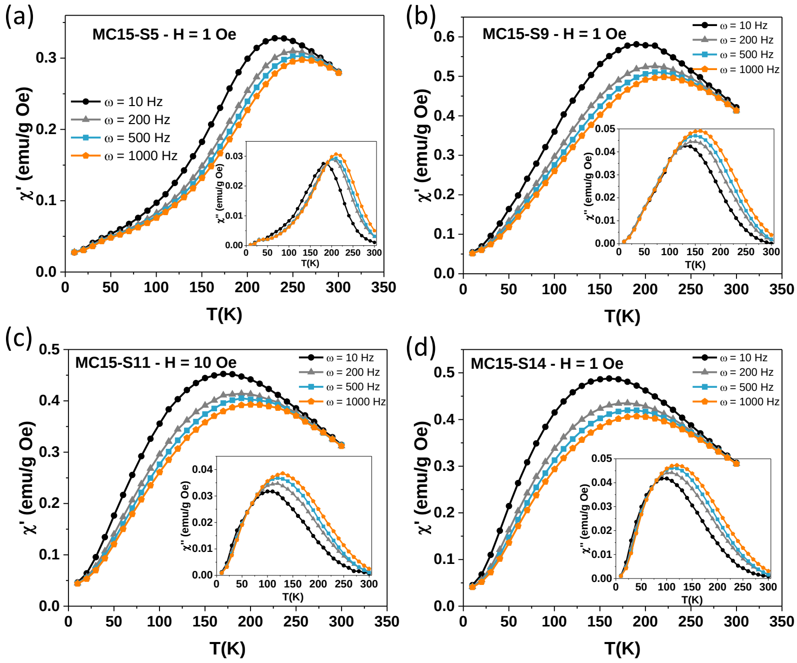

Figure S8. Temperature dependence of the real component χ'(T) and χ''(T) (inset) of **(a)** MC15-S5, **(b)** MC15-S9, **(c)** MC15-S11, **(d)** MC15-S14 MNPs at 1 Oe.

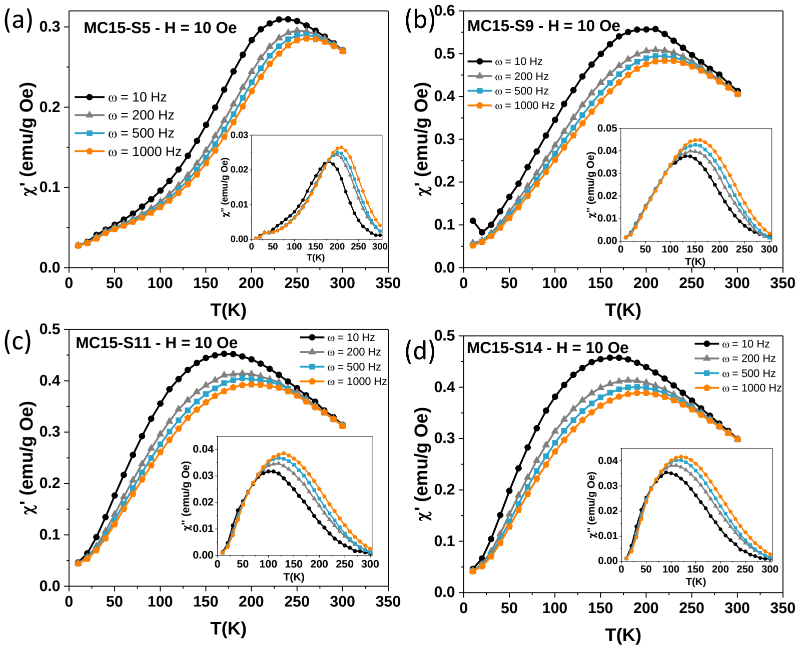

Figure S9. Temperature dependence of the real component χ'(T) and χ''(T) (inset) of **(a)** MC15-S5, **(b)** MC15-S9, **(c)** MC15-S11, **(d)** MC15-S14 MNPs at 10 Oe.

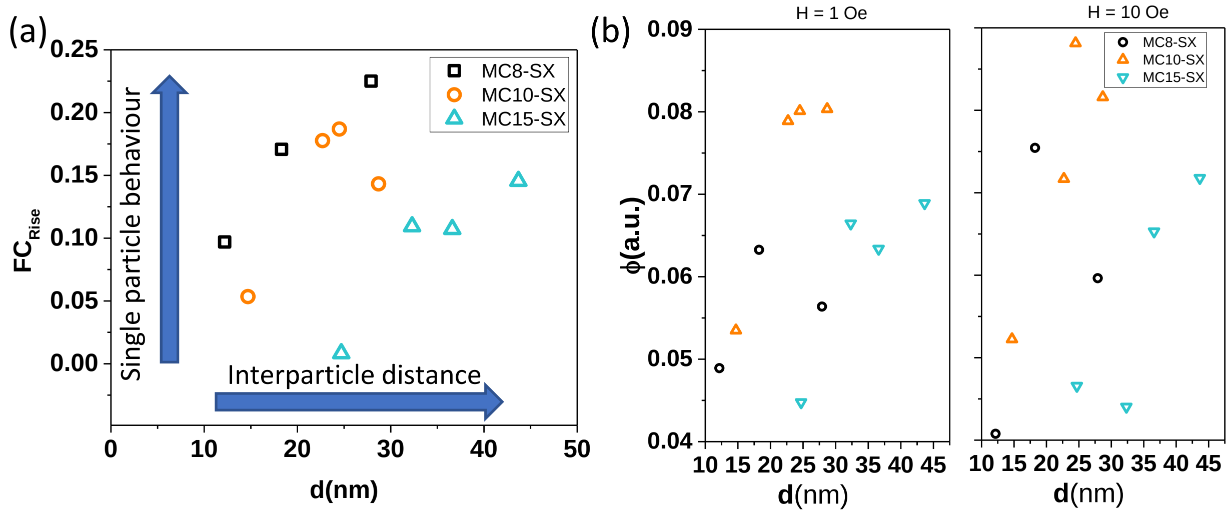

Figure S10. (a) Variation of FC_{rise}, which makes it possible to estimate the type of behaviour of dense systems of MNPs, as a function of the interparticle distance. The blue arrow on Y-axis indicates the transition from a collective to a single particle state. The blue arrow on the X-axis indicates the decrease of interactions between MNPs with increasing distance between magnetic cores. **(b)** Variation of the ϕ parameter with the distance between cores obtained through the shift in temperature with the frequency obtained by the $\chi''(T)$ curve.

IV. Blocking Temperature Determination

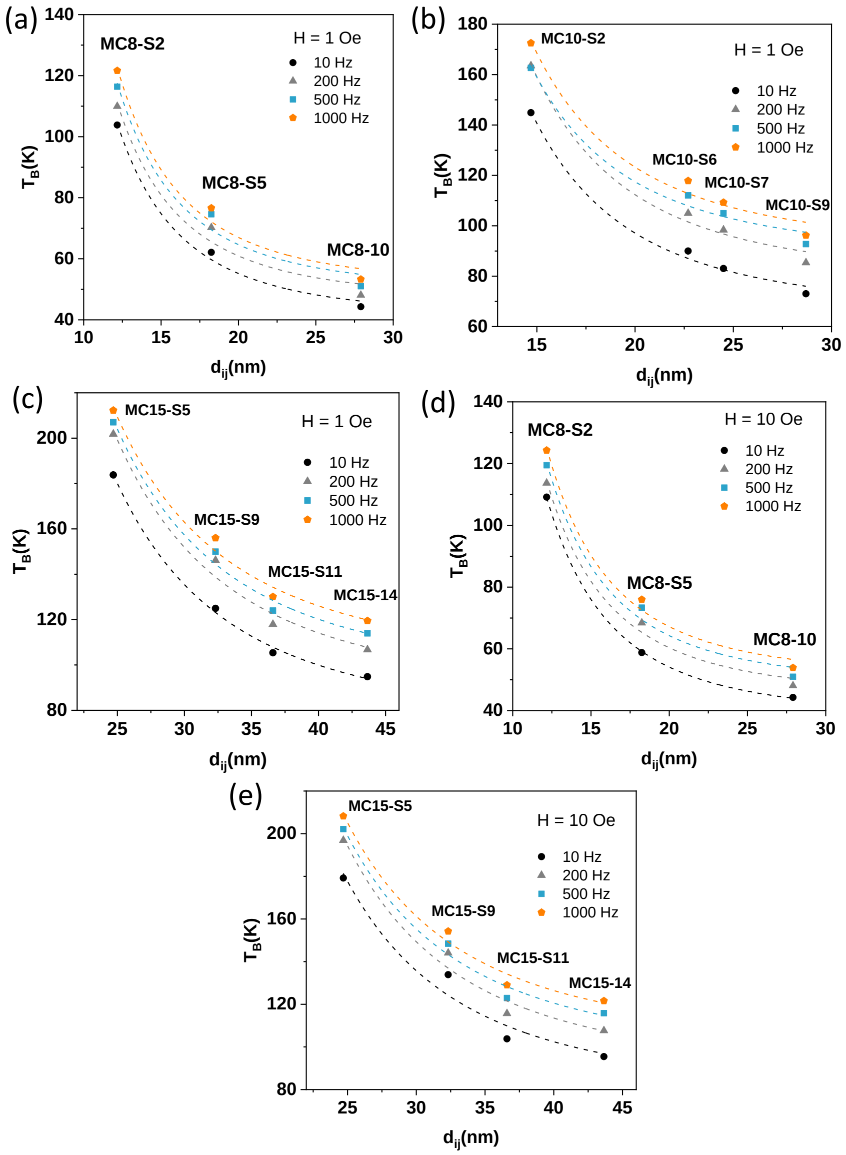

Figure S11. Blocking temperature (T_B) dependence with interparticle distance of **(a)** MC8-SX batch, **(b)** MC10-SX batch, **(c)** M15-SX batch at H=1 Oe and **(d)** MC8-SX and **(e)** M15-SX at H=10 Oe

V. Energy Barrier Determination

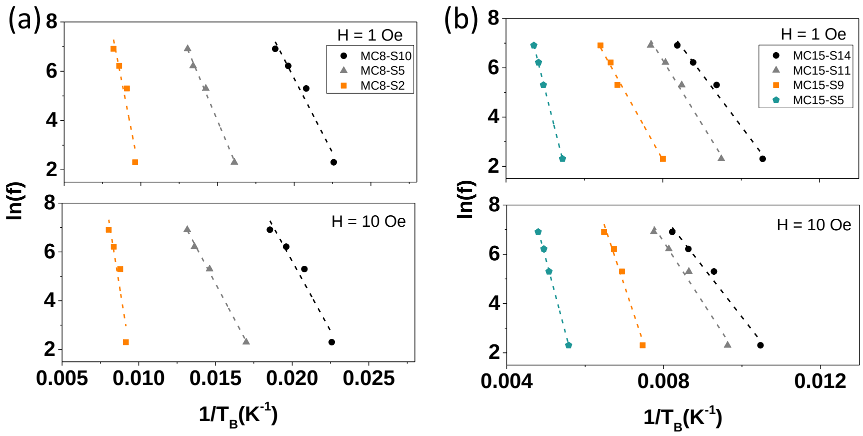

Figure S12. Semilog plot of the frequency versus the inverse of the blocking temperature of **(a)** MC8-SX MNPs and **(b)** MC15-SX MNPs at 1 Oe (top) and 10 Oe (bottom).
